# The psychological impact of COVID-19 on Chinese healthcare workers: a systematic review and meta-analysis

**DOI:** 10.1007/s00127-022-02264-4

**Published:** 2022-03-24

**Authors:** Nana Xiong, Kurt Fritzsche, Yiqi Pan, Johanna Löhlein, Rainer Leonhart

**Affiliations:** 1 Peking University Sixth Hospital, Peking University Institute of Mental Health, Beijing, China; 2 NHC Key Laboratory of Mental Health (Peking University), National Clinical Research Center for Mental Disorders (Peking University Sixth Hospital), Beijing, China; 3 Department of Psychosomatic Medicine and Psychotherapy, University Medical Centre Freiburg, Freiburg, Germany; 4 Department of Psychosomatic Medicine and Psychotherapy, University Medical Centre Hamburg-Eppendorf, Hamburg, Germany; 5 Department for Social Psychology and Methodology, Institute of Psychology, University of Freiburg, Engelbergerstr. 41, 79106 Freiburg, Germany

**Keywords:** COVID-19, Psychological impact, Mental health, Stress, Chinese healthcare workers

## Abstract

**Purpose:**

This study aimed at investigating five dimensions of the psychological impact (post-traumatic stress symptoms (PTSS), anxiety, depression, sleep disturbance or profession-related burnout) of COVID-19 on healthcare workers (HCW) in China.

**Methods:**

Studies that evaluated at least one of the five target dimensions of the psychological impact of COVID-19 on HCW in China were included. Studies with no data of our interest were excluded. Relevant Databases were searched from inception up to June 10, 2020. Preprint articles were also included. The methodological quality was assessed using the checklist recommended by AHRQ. Both the rate of prevalence and the severity of symptoms were pooled. The protocol was registered in PROSPERO (CRD42020197126) on July 09, 2020.

**Results:**

We included 44 studies with a total of 65,706 HCW participants. Pooled prevalence rates of moderate to severe PTSS, anxiety, depression, and sleep disturbances were 27% (95% CI 16%-38%), 17% (13–21%), 15% (13–16%), and 15% (7–23%), respectively; while the prevalence of mild to severe level of PTSS, anxiety, and depression was estimated as 31% (25–37%), 37% (32–42%) and 39% (25–52%). Due to the lack of data, no analysis of profession-related burnout was pooled. Subgroup analyses indicated higher prevalence of moderate to severe psychological impact in frontline HCW, female HCW, nurses, and HCW in Wuhan.

**Conclusion:**

About a third of HCW in China showed at least one dimension of psychological symptoms during the COVID-19 pandemic, whereas the prevalence of moderate and severe syndromes was relatively low. Studies on profession-related burnout, long-term impact, and the post-stress growth are still needed.

**Supplementary Information:**

The online version contains supplementary material available at 10.1007/s00127-022-02264-4.

## Introduction

The coronavirus disease 2019 (COVID-19) outbreak has rapidly spread worldwide and posed a serious public health threat. However, when the outbreak was firstly noticed in November 2019 in Wuhan, the capital of Hubei province in China, no one ever knew about this disease and the public panicked. All of the sudden, healthcare workers (HCW) in China experienced a tremendous increase in both physical workload and psychological stress [1]. Learning lessons from several past viral epidemics, such as the severe acute respiratory syndrome (SARS) and the Ebola virus disease, frontline HCW had greater levels of both acute or post-traumatic stress and general psychological distress [2]. Therefore, the mental health of HCW should be examined in COVID-19.

Fortunately, the psychological impact of COVID-19 on HCW from China has already been noticed and assessed. Generally, an increased prevalence of mental illnesses, such as post-traumatic stress disorder (PTSD), depression, and anxiety disorders was indicated, but the prevalence rates varied greatly by different studies. Several systematic reviews and meta-analyses were also conducted. Among them, a review carried out by Pappa et al. showed that the prevalence of depression, anxiety, and insomnia was 23.2%, 22.8%, and 38.9%, respectively [3]. However, the review only covered studies published in the early stages of COVID-19. A more recent meta-analysis showed that the prevalence of anxiety and depression in HCW was similar to the general public yet lower than patients with pre-existing conditions and a COVID-19 infection [4]. Nevertheless, it did not account for the fact that the criteria for case definition varied between the studies, and the pooled results could not distinguish those with only mild or subclinical syndromes from those with more severe symptoms and in need of professional help. In addition, even though most studies included were conducted in China, no paper published in Chinese was included in these systematic reviews. Moreover, the severity of psychological impact as reflected by continuous variables were never included.

Therefore, a systematic review and meta-analysis encompassing the most recent studies both published in English and Chinese is needed to investigate the psychological impact of COVID-19 on HCW in China. Besides anxiety and depression, we took more dimensions into account, such as post-traumatic stress symptoms (PTSS), sleep disturbances and profession-related burnout. Additionally, this review examined the prevalence of psychological problems by varying degrees of severity. Subsequently, subgroup analyses were conducted for gender, occupational group, location (Wuhan vs. Hubei other than Wuhan vs. other provinces), and previous working experience (frontline HCW who were defined as caring for people with confirmed or suspected COVID-19 vs. non-frontline HCW).

## Methods

The review was performed according to the Preferred Reporting Items for Systematic Reviews and Meta-Analyses (PRISMA) statement [5]. The review protocol was registered in the international prospective register of systematic reviews (PROSPERO) (CRD42020197126) on July 09, 2020.

### Data sources

Relevant records that were published until June 10, 2020 were searched in the databases of Medline, PsycINFO, EMBASE, the Cochrane Library (including Cochrane Database of Systematic Reviews), and main Chinese databases including Sinomed, the China National Knowledge Infrastructure (CNKI), and WanFang data. Preprint articles published on Medrxiv and SSRN servers, as well as the Google Scholar, and the daily updated WHO COVID-19 database were also included. The search strategy for Medline was provided in the supplementary materials. The language was restricted to English, Chinese or German. Furthermore, the reference lists from reviewed articles were searched to identify and retrieve relevant articles.

### Inclusion and exclusion criteria

We included studies that evaluated the psychological impact of COVID-19 on HCWs in China, which ought to include at least one of the following five target dimensions: PTSS, anxiety, depression, sleep disturbance or profession-related burnout. To be included into the meta-analysis, studies should use measurements that were proved to be valid to measure at least one of our target dimensions. Therefore, studies were excluded if they only measured general distress using tools of the General Health Questionnaire (GHQ-12) [6], or used non-validated self-designed questionnaire [7] or one single question of “what has been your mental attitude since COVID-19 outbreak” [8].

In order to ensure the study quality, we only included Chinese articles that were published in journals which are incorporated in the Chinese Science Citation Database (CSCD).

Both cross-sectional studies and interventional studies aligning with our criteria would be included, provided that, for the latter, the baseline level of psychological impact was extractable. Surveys investigating both HCW and other populations were included only if the data on HCW could be extracted separately.

Studies with neither data of our interest nor extractable data were excluded. When papers contained post-hoc analyses of an already included study, data was combined into one data set [9–12].

### Study quality assessment

The methodological quality of the included studies was assessed using an 11-item checklist recommended by Agency for Healthcare Research and Quality (AHRQ) [13]. An item was scored ‘0’ if it was answered ‘NO’ or ‘UNCLEAR’; if it was answered ‘YES’, then the item scored ‘1’. Article quality was assessed as follows: low quality = 0–3; moderate quality = 4–7; high quality = 8–11.

### Measurements

Primary outcomes include the prevalence of moderate to severe psychological impact, i.e. its five dimensions, PTSS, anxiety, depression, sleep disturbances, and profession-related burnout. The secondary outcomes include the prevalence of mild to severe psychological impact, and the severity of the psychological impact which were reflected through continuous variables.

The severities of each dimension were defined according to the validated cut-off values of each measure.

### Data extraction

Data extraction was then independently carried out by three authors (NNX, YP and JL). To compute the prevalence rate, both the number of confirmed cases and the total amount of participants was extracted. For primary outcomes of the prevalence of moderate to severe cases, only participants who indicated symptoms above the cut-off for moderate symptoms were classified as burdened. Participants with no or mild symptoms were classified as not burdened. Continuous outcomes were analysed using the number of participants, the mean, and the standard deviation (STD). Missing STD values were calculated from reported confidence intervals (CI), standardized errors (SE), or p values. When none of the above data were reported, study authors were contacted via e-mail for further information.

As some studies report the prevalence rates of mild, moderate, severe symptoms plus the mean and STD of the same scales, these studies were included in the pooled analyses for both primary and secondary outcomes.

### Data synthesis and statistical analysis

Statistical heterogeneity was tested using the *I*^2^ statistic and the natural approximate chi-square test [14]. *I*^2^ values above 50% indicated high heterogeneity. High heterogeneity was indicated by *p* values smaller than 0.10. The random effects model was used for heterogeneous data. The rate of prevalence was pooled by the inverse variance method. For continuous data, because of the variance in measurements across studies, the reported values were first transformed into the standardized values with a range from 0 to 100, according to the possible ranges of each questionnaire. Then the standardized values were pooled and compared between different subgroups. The likelihood of significant publication bias was assessed by both Begg’s test and Egger’s test. In addition, funnel plots were provided as a visual tool for publication bias. The Stata (15.1) [15] and the *metan* package [14] was used for statistical analyses.

### Subgroup analyses

Subgroup analyses were planned a priori to investigate potential moderators influencing the psychological distress of HCW, and thereby, to assess the sources of high heterogeneity. Therefore, both primary and secondary outcomes were compared by frontline HCW (yes/ no), gender, occupation and work location. Subgroup analysis was performed when there were at least two comparisons included. Further meta-analysis was performed in different subgroups. Meta-regressions were not performed here due to the partially small number of studies.

### Sensitivity analyses

Sensitivity analyses were conducted to test the reliability of primary outcomes, which were performed by excluding studies with low quality both individually and altogether.

## Results

### Characteristics of included studies

**Fig. 1:**
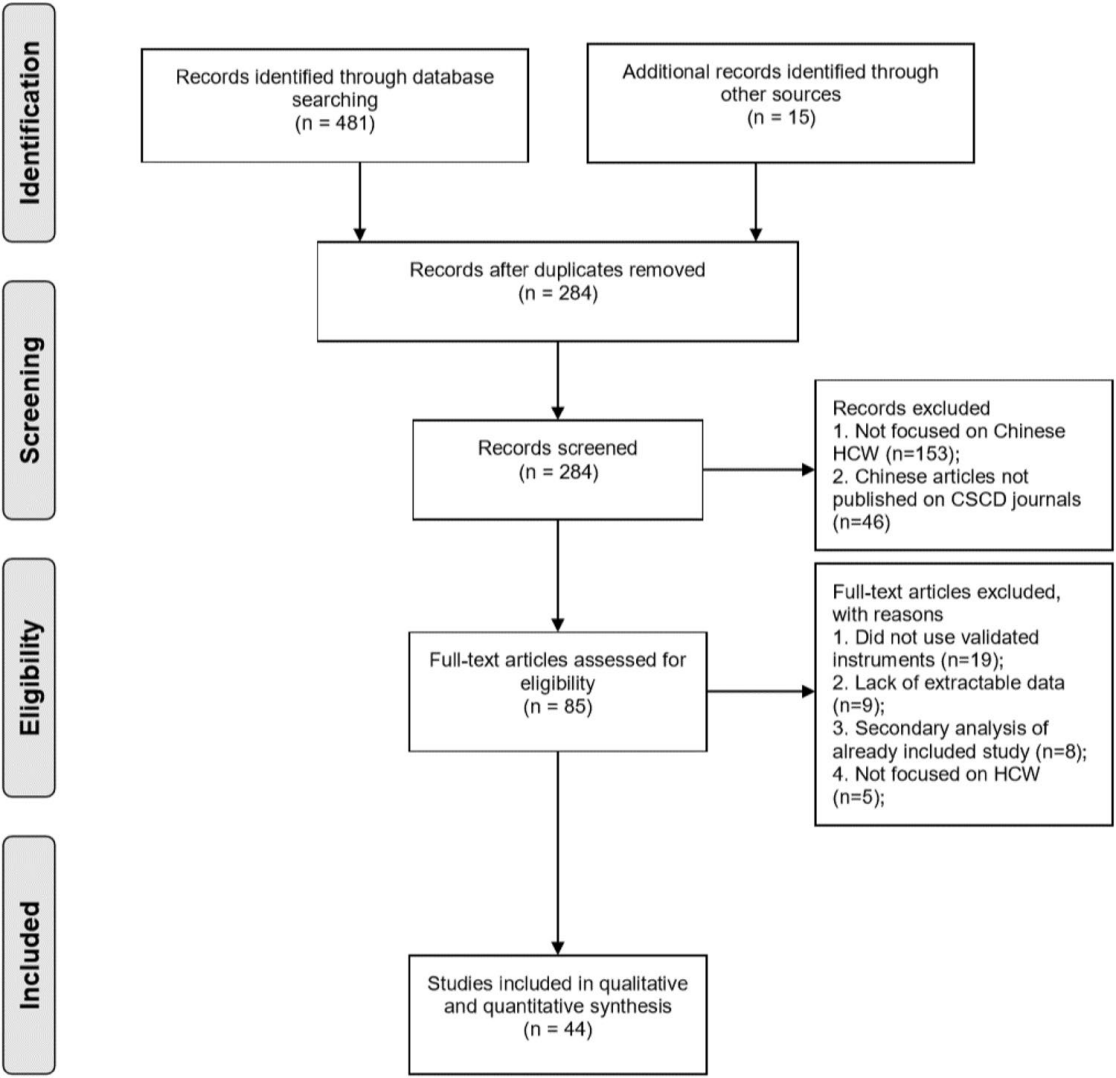
PRISMA Flow diagram of the study selection process

Overall, 85 full text articles were obtained and assessed for eligibility (Fig. 1). Of these, 44 studies met the inclusion criteria for the review, and were included in the meta-analysis [10, 11, 16–57].

44 studies with a total of 65,706 participants were included (see Table 1), with 76.7% being women. Apart from the studies that did not report the specific occupation of HCW, a total of 19,316 (33.0%) doctors, 35,644 (60.9%) nurses and 3552 (6.1%) technicians and administrative staff were investigated. As shown in Table 1, most studies were conducted from early to mid-February, at the height of the COVID-19 epidemic in China. Regarding the locations of the studies, 12 were conducted in Wuhan city, 3 in Hubei province, 16 in other provinces in China, and another 13 at multiple centres nationwide or in unknown areas. Twenty-four of the included studies were published in English, whereas the remaining 20 studies were published in Chinese. No articles in German were identified.

**Table 1:** Overview of the characteristics of included studies (organized by regions)

Author	Date (in 2020)	Sample size	Response rate (%)	HCW subgroup (%)			Female (%)	Age (SD)	Prevalence of psychological impact** (%)				Quality score (AHRQ)
				Doctor	Nurse	Others			PTSS	Anxiety	Depression	Insomnia	
*Region: Wuhan city*													
Du 2020	2.13–2.17	134	43.2	35.1	41.0	23.9	60.5	36.0 (8.1)	–	20.1*	12.7*	–	5
Jiang 2020	1.27–2.1	175	UNK	–	100	–	100	31.7 (5.9)	–	–	–	–	5
Li 2020a	2.8–2.11	205	UNK	–	100	–	85.4	UNK	50.7	–	–	–	6
Li 2020b	Jan–Feb	66	100	–	100	–	77.3	UNK	–	–	–	–	5
Li 2020c	UNK	121	100	–	100	–	89.3	30.3 (5.4)	–	–	–	–	5
Li 2020d	2.8–2.15	4369	88.2	13.3	77.4	9.3	100	UNK	31.6	25.2	14.2	–	6
Li 2020e	2.17–2.21	526	UNK	–	100	–	85.6	29.5 (5.0)	–	–	–	–	3
Mo 2020	2.21	180	85.7	UNK	UNK	UNK	90.0	32.7 (6.5)	–	–	–	–	5
Wang 2020	UNK	112	UNK	UNK	UNK	UNK	76.8	34.3 (6.1)	–	–	–	–	2
Xu 2020a	UNK	41	UNK	–	100	–	90.2	31.3 (2.5)	–	–	–	–	4
Yuan 2020	2.7–2.20	309	85.6	43.7	48.5	7.8	76.4	34.0 (8.9)	–	–	–	–	4
Zhu 2020b	2.8–2.10	5062	77.1	19.8	67.5	12.7	85.0	UNK	29.8	24.1	13.4	–	8
*Region: other cities in the Hubei province*													
Qi 2020	Feb	1306	UNK	UNK	UNK	UNK	80.4	33.1 (8.4)	–	–	–	71.7*	7
Xing 2020	1.22	40	UNK	–	100	–	95.0	31.4 (7.3)	–	–	–	–	4
Xu 2020b	2.9	42	100	–	100	–	100	UNK	–	35.7	–	–	5
*Region: outside of the Hubei province*													
Cai 2020	2.1–2.7	1521	UNK	33.6	35.9	11.9	75.5	UNK	–	–	–	–	3
Cao 2020	UNK	37	100	43.2	56.8	–	78.4	32.8 (9.6)	–	–	18.9	–	4
Chen 2020	UNK	206	93.7	84.0		16.0	69.5	34.0(8.9)	–	0.5	–	–	3
Deng 2020a	UNK	60	UNK	30.0	70.0	–	86.7	32.2 (8.0)	–	–	–	–	3
Deng 2020b	Jan	68	100	UNK	UNK	UNK	66.2	UNK	–	–	–	–	3
Huang 2020a	2.7–2.14	230	93.5	30.4	69.6	–	81.3	32.6 (6.2)	27.4*	7.0	–	–	6
Liang 2020	2.3–2.21	59	UNK	39.0	61.0	–	UNK	UNK	–	–	–	–	2
Liu 2020a	2.1–2.18	1097	100	–	100	–	98.3	29.0 (5.9)	–	3.5	9.7	2.8	3
Luo 2020	2.10–2.20	171	91.9	UNK	UNK	UNK	76.0	UNK	–	15.2	17.5	–	6
Pu 2020	UNK	867	UNK	–	100	–	95.6	30.8 (7.1)	19.3	–	–	–	3
Sheng 2020	UNK	92	96.8	–	100	–	93.5	21.3 (1.0)	–	–	–	17.4*	4
Tian 2020	4.6–4.10	845	79.9	23.2	76.8	–	84.5	35.5 (6.7)	–	20.7*	45.6*	27.0*	4
Wu 2020a	UNK	106	94.6	–	100	–	80.2	30.8 (4.5)	–	29.3	–	64.2*	4
Wu 2020b	UNK	120	UNK	UNK	UNK	UNK	74.2	33.7 (12.2)	–	–	–	–	3
Yin 2020	2.3–2.5	1266	UNK	UNK	UNK	UNK	74.7	35.2 (7.8)	–	11.7	10.2	–	8
Zhu 2020a	2.1–2.29	165	100	47.9	52.1	–	83.0	34.2 (8.1)	–	20.0*	44.2*	–	6
*Region: multiple centers nationwide or unknown*													
Guo 2020	2.18–2.20	11,118	UNK	30.3	53.1	16.6	74.8	UNK	–	5.0	13.5	–	3
Huang 2020b	2.3–2.17	2250	UNK	UNK	UNK	UNK	UNK	UNK	–	35.6	19.8	23.6	4
Huangfu 2020	UNK	326	100	–	100	–	100	28.6 (12.2)	–	–	–	–	3
Lai 2020	1.29–2.3	1257	68.7	39.2	60.8	–	76.7	UNK	35.0	12.3	14.8	7.8	6
Liu 2020b	2.10–2.20	512	85.4	UNK	UNK	UNK	84.6	UNK	–	2.1	–	–	4
Liu 2020c	2.17–2.24	4679	UNK	39.6	60.4	–	82.3	35.9 (9.0)	–	5.2	19.8	–	4
Lv 2020	UNK	7071	UNK	52.2	47.8	–	71.2	UNK	–	35.6*	37.0*	35.2*	3
Song 2020	2.28–3.18	14,825	UNK	41.1	58.9	–	64.3	34.0 (8.2)	7.3	–	15.2	–	3
Sun 2020	1.31–2.4	442	UNK	12.0	78.7	9.3	83.3	UNK	–	–	–	–	5
Xiao 2020a	Jan–Feb	180	81.8	45.6	54.4	–	71.7	32.3 (4.9)	–	36.7	–	31.7	4
Xiao 2020b	1.28	958	UNK	39.5	37.5	23.1	67.2	UNK	–	54.1*	57.3*	–	2
Zhang 2020a	2.19–3.6	927	UNK	UNK	UNK	UNK	73.1	UNK	–	13.0	12.2	10.5	3
Zhang 2020b	1.29–2.3	1563	UNK	29.0	63.0	8.0	82.7	UNK	37.4	12.9	17.2	–	5

*AHRQ* Agency for Healthcare Research and Quality, *HCW* healthcare workers, *PTSS* post-traumatic stress symptoms, *UNK* unknown

Prevalence of psychological impact**: the prevalence of moderate to severe level of burden was presented if available; if not, the prevalence of mild to severe level of burden was used with a mark of *. For all studies with no prevalence indicated, only the severity data was available and extracted

The total quality score is added up from all applicable items (potential range: 0–11). Item 1: source of information defined; Item 2: clear criteria for exposed and exposed subjects; Item 3: time period for identifying patients indicated; Item 4: whether or not subjects were consecutive if not population-based indicated; Item 5: whether subjective components of study were masked to other aspects of the status of the participants indicated; Item 6: quality assurance for assessments undertaken; Item 7: patient exclusions from analysis explained; Item 8: confounding assessment or/and control described; Item 9: missing data handling explained (if existent); Item 10: response rates and completeness of data collection indicated; Item 11: expected follow-up clarified (if any)

All selected articles were assessed for methodological quality. According to the criteria of AHRQ, only two studies were of high quality, 26 studies were of moderate quality, and 16 studies were of low quality (see Table 1 and detailed information in Supplementary Table 1).

All assessment tools and the according cut-off values employed to measure the psychological impact are listed in Table 2.

**Table 2:** Overview of the measurements employed to assess the psychological impact of COVID-19 on HCW in China

Measurements	Prevalence criteria^a^			Studies
*PTSS*	At least Moderate		At least mild	
Impact of Event Scale- Revised (IES-R)	≥ 26		≥ 9	Lai 2020; Li 2020b; Sun 2020a; Zhang 2020b; Zhu 2020b
PTSD Checklist Civilian Version (PCL-C)	> 38		NA	Li 2020a; Wu 2020b
Post-Traumatic Stress Disorder Self-Rating Scale (PTSD-SS)	NA		50	Huang 2020b
PTSD Checklist for DSM-5 (PCL-5)	> 38		> 33	Song 2020
Triage Assessment Form (TAF)	≥ 13		NA	Pu 2020
Stanford Acute Stress Reaction Questionnaire (SASRQ)	Severity only			Xiao 2020a
Vicarious Traumatization Questionnaire (VTQ)	Severity only			Li 2020e
*Anxiety*				
Zung Self-Rating Anxiety Scale (SAS)	≥ 60	≥ 50		Chen 2020a; Guo 2020; Huang 2020b; Liang 2020; Liu 2020a; Liu 2020d; Mo 2020; Pu 2020; Sheng 2020; Wu 2020a; Wu 2020b; Xiao 2020a; Xu 2020; Zhu 2020a
Generalized Anxiety Disorder 7-item (GAD-7)	≥ 10	≥ 5		Huang 2020a; Lai 2020; Li 2020b; Li 2020d; Liu 2020c; Luo 2020b; Lv 2020; Tian 2020; Zhang 2020a; Zhang 2020b; Zhu 2020b
Symptom Checklist 90; Anxiety subscale (SCL-90)	Severity only			Cai 2020b; Deng 2020b; Huangfu 2020; Jiang 2020; Wang 2020b; Xing 2020; Xu 2020
Hamilton Anxiety Rating Scale (HAM-A)	≥ 21	≥ 14		Li 2020c
Stress-related Questions associated with the H1N1 event; Anxiety subscale	Severity only			Deng 2020a
Beck Anxiety Inventory (BAI)	NA	≥ 8		Du 2020
Hospital Anxiety and Depression Scale; Anxiety subscale (HADS)	NA	≥ 8		Xiao 2020b
*Depression*				
Patient Health Questionnaire 9-item (PHQ-9)	≥ 10	≥ 5		Cao 2020; Lai 2020; Li 2020b; Li 2020d; Liu 2020c; Luo 2020b; Lv 2020; Tian 2020; Zhang 2020a; Zhang 2020b; Zhu 2020b
Symptom Checklist 90; Depression subscale (SCL-90)	Severity only			Cai 2020b; Deng 2020b; Huangfu 2020; Jiang 2020; Wang 2020b; Xing 2020; Xu 2020
Zung Self-Rating Depression Scale (SDS)	≥ 60	≥ 50		Guo 2020; Liang 2020; Liu 2020d; Sheng 2020; Wu 2020a; Zhu 2020a
Center for Epidemiology Studies Depression Scale (CES-D)	> 28			Huang 2020a; Song 2020
Beck Depression Inventory (BDI-II)	NA	≥ 14		Du 2020
Hospital Anxiety and Depression Scale; Depression subscale (HADS)	NA	≥ 8		Xiao 2020b
*Sleep disturbance*				
Pittsburgh Sleep Quality Index (PSQI)	≥ 11	≥ 6		Huang 2020a; Li 2020d; Qi 2020; Sheng 2020; Wu 2020a; Wu 2020b; Xiao 2020
Insomnia Severity Index (ISI)	≥ 15	≥ 8		Lai 2020; Liu 2020c; Lv 2020; Tian 2020; Zhang 2020a; Zhang 2020b
Athens Insomnia Scale (AIS)	NA	> 6		Qi 2020
*Profession-related burnout*^b^				
Maslach Burn-out Inventory (MBI)	NA			Cao 2020; Wu 2020c
Stress-related questions associated with the H1N1 event; Burnout subscale	Severity only			Deng 2020a

*PTSS* post-traumatic stress symptoms, *NA* not applicable

^a^Data extraction was conducted separately for the primary outcome (% prevalence for at least moderate symptoms), and for the secondary outcomes (% prevalence for at least mild symptoms; Severity of symptoms). Studies with pertinent information for at least one outcome were included in the meta-analysis. Studies which indicated data for both primary outcomes and secondary outcomes were both included in the respective pooled analyses

^b^Profession-related burnout was not pooled since the original papers did not report the prevalence rates, or the case definition used did not align with the common criteria

### Primary outcome: prevalence of moderate to severe levels of psychological impact

#### PTSS

**Fig. 2:**
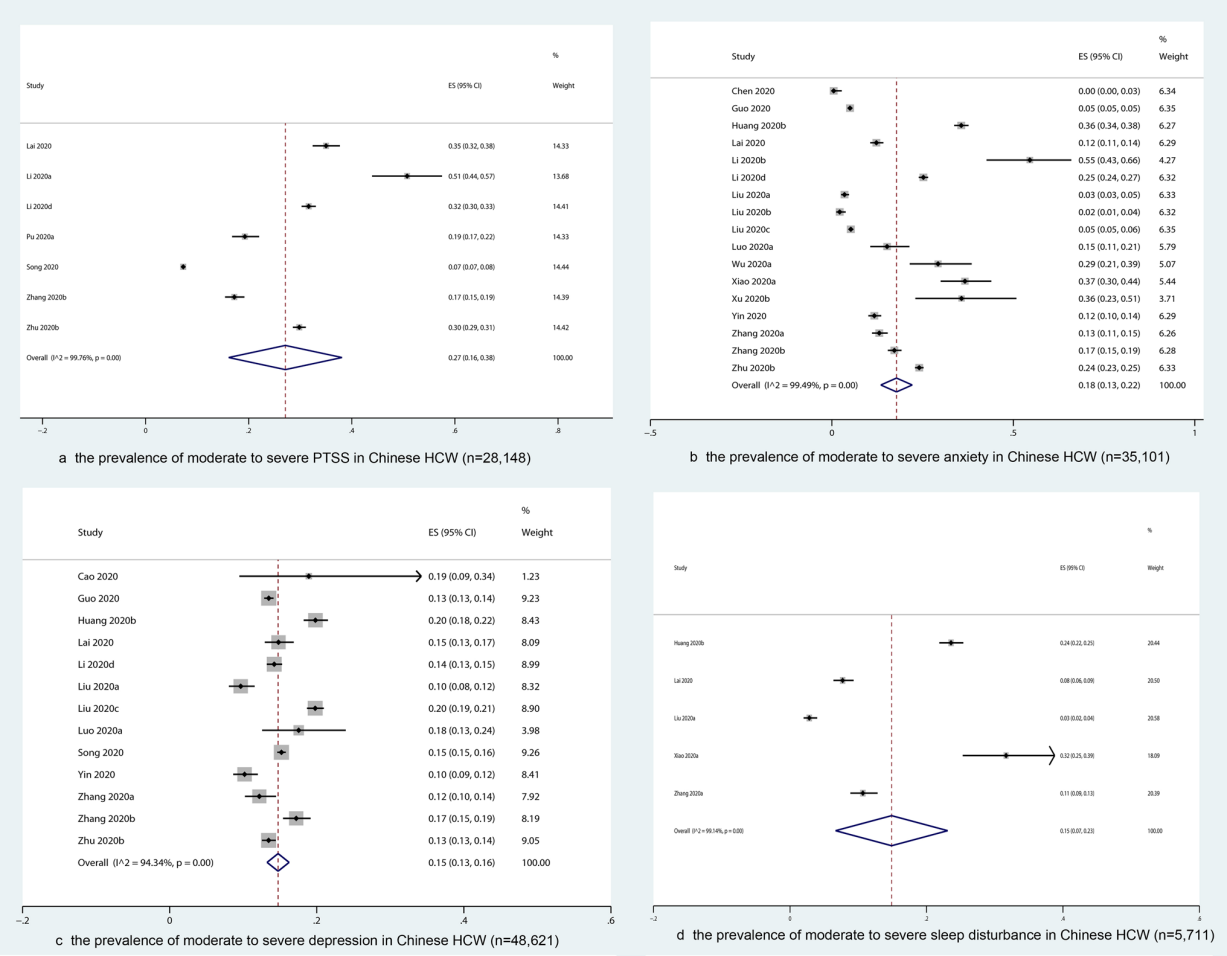
The prevalence of moderate to severe psychological impact on the whole sample of Chinese HCW

Seven studies with 28,148 participants measured the prevalence of moderate to severe PTSS (Fig. 2a). The overall prevalence was 27% (95% CI 16–38%) with substantial heterogeneity (*I*^2^ = 99.8%, *p* = 0.02).

**Fig. 3:**
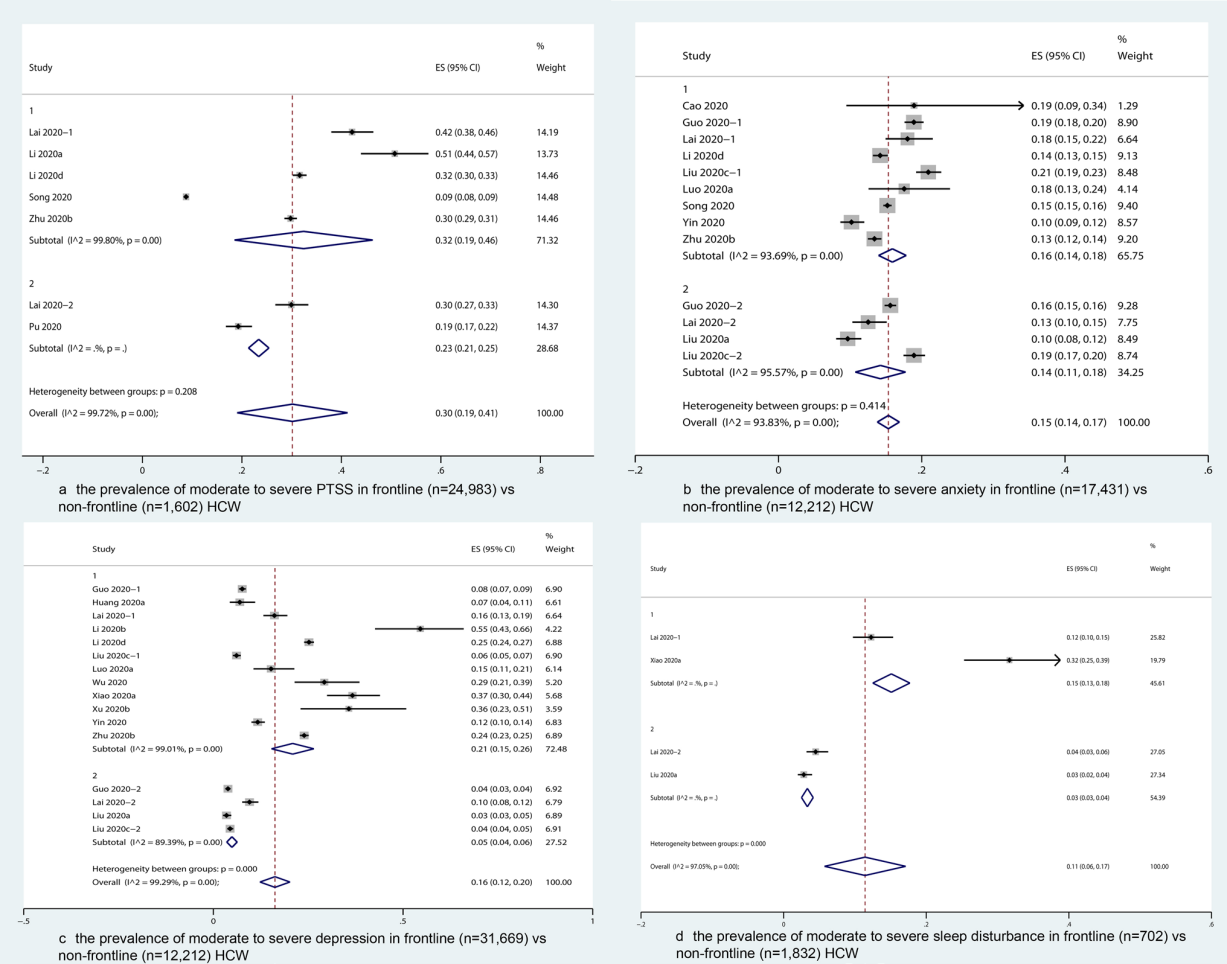
The prevalence of moderate to severe psychological impact on frontline vs. non-frontline HCW

Among them, six studies (seven subgroups) reported the prevalence for frontline and non-frontline HCWs separately. Subgroup analysis showed that the prevalence of moderate to severe PTSS was higher in frontline [32% (95% CI 19–46%)] than non-frontline HCW [23% (95% CI 21–25%)] (Fig. 3a).

Additional subgroup analyses showed that the prevalence of moderate to severe PTSS was significantly higher in female [33% (95% CI 31–35%)] than male HCW [22% (95% CI 20–25%)], was significantly higher in nurses [34% (95% CI 28–40%)] than technicians or others [22% (95% CI 20–25%)], and was significantly higher in HCW from Wuhan [36% (95% CI 32–41%)] than those from other provinces in China [21% (95% CI 18–23%)] (Supplementary Figs. 1–3).

The difference between gender, occupations and locations could partly account for the large heterogeneity (*p* < 0.01).

#### Anxiety

Resulting from 18 studies with 34,793 participants, the overall prevalence of moderate to severe anxiety was estimated as 17% (95% CI 13–21%) (Fig. 2b).

Subgroup analysis showed that the prevalence of moderate to severe anxiety was higher in frontline [21% (95% CI 15–26%)] than non-frontline participants [5% (95% CI 4–6%)] (Fig. 3b), and higher in HCW from Wuhan [25% (95% CI 20–30%)] than from other cities in the Hubei province [9% (95% CI 6–13%)], and other provinces in China [10% (95% CI 5–14%)], but was comparable in female and male participants, as well as in nurses, doctors, and technicians (Supplementary Figs. 4–6).

The difference between positions (frontline vs. non-frontline) and locations could partly account for the large heterogeneity within the whole sample (*p* < 0.01).

#### Depression

The prevalence of moderate to severe level of depression was estimated as 15% (95% CI 13–16%) (*k* = 13, *n* = 48,621) (Fig. 2c).

Subgroup analysis showed that the prevalence of moderate to severe depression was comparable between frontline and non-frontline HCW (Fig. 3c), and between female and male HCW. In contrast, the prevalence was significantly higher in nurses [17% (95% CI 14–21%)] than technicians or others [11% (95% CI 8–13%)], and significantly higher in HCW from Wuhan [14% (95% CI 13–16%)] than those from other provinces in China [11% (95% CI 9–13%)] (Supplementary Figs. 7–9).

Only the differences between locations could partly account for the heterogeneity (*p* = 0.04).

#### Sleep disturbance

The overall prevalence of moderate to severe sleep disturbance was estimated as 15% (95% CI 7–23%, *k* = 5, *n* = 5,711) with substantial heterogeneity (*I*^2^ = 99.1%, *p* = 0.01) (Fig. 2d). As indicated in Fig. 3d, sleep disturbance was significantly more severe among frontline [15% (95% CI 13–18%)] than non-frontline HCW [3% (95% CI 3–4%)]. Such subgroup difference could partly account for the heterogeneity (*p* < 0.01). Due to the lack of data, no subgroup analysis on gender, occupational group* and location was conducted.

### Secondary outcomes: prevalence of mild to severe levels of psychological impact

The prevalence of mild to severe PTSS was not pooled due to the lack of data. The prevalence of mild to severe levels of anxiety, depression and sleep disturbances was estimated as 31% (95% CI 25–37%), 37% (95% CI 32–42%) and 39% (95% CI 25–52%), respectively (Supplementary Figs. 10–12).

### Secondary outcomes: severity of psychological impact

Reflected by continuous data, the psychological impact was pooled and compared between different subgroups. Take the pooled ES of both frontline and non-frontline HCW for an example, on a standardized range between 0 and 100, all dimensions of psychological impact, including PTSS [SMD = 37.0 (95% CI 28.2–45.8)], anxiety (SMD = 43.7 (95% CI 39.6–47.8)], depression [SMD = 37.4 (95% CI 29.4–45.5)], and sleep disturbances [SMD = 36.2 (95% CI 25.1–47.3)] fall into a mild to moderate range.

#### Severity of psychological impact in frontline vs. non-frontline HCW

We found no significant differences between frontline and non-frontline HCW regarding the severity of PTSS, anxiety, and depression. However, sleep disturbance was more severe in frontline HCWs [SMD = 43.2 (95% CI 33.1–53.3) vs. 25.5 (95% CI 14.8–36.2)] (Supplementary Figs. 13–16).

#### Severity of psychological impact in female vs. male HCW

Based on the pooled ES, no significant differences were found between female and male HCW in terms of the severity of PTSS, anxiety, depression, as well as sleep disturbances (Supplementary Figs. 17–20).

#### Severity of psychological impact on HCWs with different occupations

Due to the lack of data, comparisons were only conducted between doctors and nurses. Our results indicate that the severity of PTSS, anxiety, depression, and sleep disturbance appear more severe in nurses, although no significant difference were given (Supplementary Figs. 21–24).

#### Psychological impact on HCWs from different locations

As the pooled ES showed, the severity of PTSS and sleep disturbance of HCW in Wuhan seemed to be higher than those from other provinces in China, but no significant difference was detected (Supplementary Figs. 25–26).

Interestingly, both levels of anxiety [SMD = 49.4 (95% CI 43.1–55.7)] and depression [SMD = 44.8 (95% CI 40.9–48.8)] were significantly higher in HCW from other cities in the Hubei province than in those from Wuhan [SMD = 32.8 (95% CI 24.0–41.5] and 28.2 (95% CI 20.2–36.2)] (Supplementary Figs. 27–28).

### Sensitivity analyses

By excluding all studies with low methodological quality, the prevalence of moderate to severe PTSS, anxiety, depression, and sleep disturbance was slightly higher, which was estimated as 32% (95% CI 26–39%), 21% (95% CI 15–27%), 16% (95% CI 14–18%), and 21% (95% CI 8–34%). The heterogeneity within each outcome remained substantial, with the *I*^2^ lowered by 0–1.6%.

### Publication bias

Results from Egger’s test showed potential publication bias concerning the prevalence of moderate to severe PTSS (*p* = 0.009), anxiety (*p* = 0.003), and depression (*p* < 0.001), but the Begg’s test did not reveal risk of publication bias in the prevalence of PTSS (*p* = 0.23). Neither test indicated potential risk in the prevalence of sleep disturbances (*p* = 0.254 and 0.221), or the severity of PTSS (*p* = 0.640 and 0.436), anxiety (*p* = 0.079 and 0.315), depression (*p* = 0.616 and 0.767) or sleep disturbances (*p* = 0.366 and 0.734) as reflected in continuous data. Funnel plots were provided in the Supplementary Figs. 29–36.

## Discussion

This systematic review and meta-analysis focused on the psychological burden of HCW in China during the COVID-19 pandemic. Compared to other reviews [3, 4, 58–60], this review covers a longer period until June 2020 and has included good quality studies in Chinese language. Additionally, this study distinguished between differing severities of psychological problems and synthesized data measured with continuous scales.

### Summary of the main findings

Forty-four studies with a total of 65,706 HCW were included. The period of studies spanned the end of January to early April 2020, at the height of the COVID-19 epidemic in China. Despite the great heterogeneity of the studies, there is strong evidence that about a third of the clinic staff showed at least one dimension of psychological symptoms. More pronounced symptoms like moderate and severe level of PTSS, anxiety, depression, and sleep disturbances, were found in 27%, 17%, 15%, and 15% of the participants examined, respectively.

### Comparison with other reviews and studies in other countries

Compared with results of the latest China mental health survey [61], the psychological burden was significantly elevated. However, the increased values differed little from the values reported for the general population in China in the above-mentioned months [4, 62, 63].

Compared to studies from other countries: a multi-centered study in Singapore and India investigated frontline HCW. In this study, only 2.2% were screened positive for moderate to extremely-severe stress, 8.7% for anxiety, and 5.3% for depression. However, the prevalence of physical discomforts was as high as 33.4% [64]. It was speculated that somatic symptoms were used to represent emotions in this situation. A study conducted during an early peak of COVID-19 in New York City also found very high positive screens for psychological symptoms as follows: 57% for acute stress, 48% for depressive symptoms, and 33% for anxiety symptoms [65].

In addition, after the data of our meta-analysis were collected, similar studies were piled up and have provided further evidence in professional-related burnout and post-traumatic growth. For example, a study showed that the burnout thresholds in disengagement and exhaustion were met by 79.7% and 75.3% of respondents [66]. Another large-scale survey of frontline nurses in China reported that 13.3% of them experienced trauma, and 39.3% experienced post-traumatic growth [67]. In a tertiary hospital of a highly burdened area of north-east Italy, 38.3% HCW showed high emotional exhaustion and 46.5% showed low professional efficacy [68].

### Relevance of key subgroups

Similar to our results considering the prevalence of psychological burden, previous evidence from China, Germany, and worldwide has shown that mental problems were more pronounced in female participants, nurses, and frontline health professionals, especially those in the departments for infectious diseases, fever clinics and intensive care units, which was in line with their exposure level and proximity to COVID-19 patients [3, 10, 58].

However, contrary to our expectations, the subgroup comparison results differed between the binary and the continuous outcomes, i.e. reflected by continuous data, basically all subgroups were comparable, and that HCW in the Hubei province even had higher levels of anxiety and depression than those in Wuhan city. One possible explanation was that since most HCW did not have severe symptoms, so that the effect sizes from higher percentages of confirmed cases were diluted. Such results also remind us to not neglect the mental health of “non-frontline” healthcare providers. In addition, during that period, it is possible that Wuhan got most of the assistance at the very beginning, and healthcare providers in other cities of the Hubei province, like Huanggang city, were suffering under more severe anxiety and depression [69].

### Comparisons with other viral epidemics

Similar to the COVID-19, several viral epidemics have occurred in the past 20 years, such as the SARS, the A/H1N1 influenza pandemic, the Middle East respiratory syndrome (MERS), and the Ebola virus disease. According to a review, in these large-scale viral outbreaks, HCW at high risks of exposure also had greater levels of both acute or posttraumatic stress (OR 1.71) and psychological distress (OR 1.74) [2]. Another similar review found that 11–73.4% of HCW reported PTSS during outbreaks, whereas depressive symptoms were reported in 27.5–50.7%, insomnia symptoms in 34–36.1%, and severe anxiety symptoms in 45% of HCW [70].

Compared with these results, our findings on the prevalence of psychological symptoms, especially the moderate and severe cases, in HCW from China under the COVID-19 were at the lower end. A possible explanation for the lower psychological distress of HCW in China could be the relatively low mortality rate, the quick control of the epidemic in China and available experiences acquired from previous pandemics, like the SARS in 2003.

### Interventions and lessons learnt

In response to the crisis, as early as Jan 27, 2020, the National Health Commission of China published a national guideline of psychological crisis intervention for COVID-19 to provide multifaceted psychological protection of the mental health of medical workers [71]. Mental health experts across the country responded quickly to form psychosocial crisis intervention teams, and offered both online and face-to-face psychological counseling, hotline services, and online platforms with psychological self-help information, such as mindfulness and relaxation techniques [69]. Based on previous experiences, they were also expected to look after the needs of teams that were newly formed in the course of the Corona-related restructuring or that have come into conflict situations due to stress overload.

However, the implementation of psychological intervention services encountered obstacles, as Chen et al. pointed out [72]. Even though medical staff showed signs of psychological distress, they denied problems and refused psychological help. Therefore, interventions were adjusted to focus more on fulfilling their basic needs, such as providing more places to rest, guaranteeing food and daily living supplies [72]. According to existing evidence, prevention efforts such as screening for mental health problems should be provided in a proper way [73].

In some studies, psychological support demonstrated protective effects. For example, the Balint group, which was developed by Michael and Enid Balint, is a small group of clinicians who meet regularly to discuss cases from their practices, with a focus on the doctor–patient relationships. In Iran, the Balint groups were found to help healthcare workers to better cope with psychosocial stressors by improving participants' insight into their experience and by facilitating group learning on the doctor-patient relationships [74].

### Limitations

Our review has several limitations. First, we only focused on studies conducted in China. Future reviews with studies from other countries that are affected by the virus at a later time period may show different exposure profiles. In particular, different health care systems will have an influence on the severity of mental stress and coping strategies. Second, the heterogeneity of the studies was high, perhaps due to the different assessment scales and its respective cut-off scores. We have tried to compensate for this heterogeneity by differentiating the moderate and severe cases from other subclinical symptoms, as well as by examining the severity reflected through continuous data. Subgroup analyses were also carried out and revealed that the frontline work, gender, occupation, and location could partly explain the high heterogeneity among the different studies. Third, even though we tried to include more high-quality studies by setting criteria for the journals in which they were published, the methodological quality of most studies was assessed as being moderate, and even low. For one, given the special background, most of the studies were carried out online so that no response rate could be provided, and the representative of the sample could not be guaranteed. Moreover, risk factors were seldom inquired, such as previous mental disorders or stressful live events, which could be the confounding factors of the current psychological distress. In addition, we did not set a criterion for the minimum time period when assessing the study quality, even though the AHRQ checklist was used to assess whether the study period was reported. It is also one of our limitations that we did not look up the subsequent studies that have referenced the studies included. Last, our analyses showed that a publication bias was likely, pertaining to the prevalence of PTSS, anxiety, and depression.

### Future research directions

Future high-quality research remains necessary to explore the impact of COVID-19 on profession-related burnout of HCW, its long-term impact, and post-stress or post-traumatic growth, i.e., the positive psychological change experienced after the struggle with COVID-19, such as to identify meaning in interpersonal relationships, to change priorities, and to have a richer spiritual life.

To improve the methodological quality, researchers should pay further attention when designing the study, and should especially consider how to improve the representativeness of the sample, and the validity and reliability of assessment tools, and how to assess and control for potential confounding factors more sufficiently.

## Conclusions

In summary, during the COVID-19 pandemic, an increased level of anxiety, depression, sleep disorders, and PTSS symptoms was detected among healthcare professionals in China. Among them, about a third showed at least mild symptoms, while moderate and severe syndromes were relatively low. Despite the low severity of the symptoms, these subsyndromal disorders should be detected and treated in time to prevent the development of complex disorders such as PTSD and to prevent the chronification of depression, anxiety and sleep disorders. In the future, high-quality studies on profession-related burnout, long-term impacts and the post-stress growth are still needed.

## Supplementary Information

Below is the link to the electronic supplementary material.

Supplementary file1 (DOCX 2818 KB)

## Data Availability

Available at request.
