# Supplementary material for: The psychological impact of COVID-19 on Chinese healthcare workers: a systematic review and meta-analysis
Source: Soc Psychiatry Psychiatr Epidemiol. 2022 Mar 24;57(8):1515–29. doi: 10.1007/s00127-022-02264-4 (PMC8943357; doi:10.1007/s00127-022-02264-4)
Supplement: Supplementary file 1 — Supplementary file1 (DOCX 2818 KB) [file 127_2022_2264_MOESM1_ESM.docx]

Search strategy for Medline:

(((((psychological* OR mental* OR stress OR anxiety OR depression OR trauma OR sleep OR burnout)) AND (healthcare worker OR medical*)) AND (China OR Chinese)) AND (COVID-19 OR Corona*)) AND (English[Language] OR Chinese[Language] OR German[Language])

Supplementary table 1. The detailed methodological quality of the included studies based on AHRQ Assessment Forms for cross-sectional studies

| Author | Item 1:  source of information | Item2:  criteria for exposure | Item3: time period | Item4: sampling: consecutive or population-based | Item5: subjective assessments were masked | Item6: quality assurance of assessments | Item 7: patient exclusions from analysis explained | Item8: confounding assessment | Item9: missing data handling explained | Item10:  response rates and completeness of data | Item11: follow-up | Total  score |
| --- | --- | --- | --- | --- | --- | --- | --- | --- | --- | --- | --- | --- |
| Cai 2020 | 1 | 0 | 1 | 0 | 0 | 1 | 0 | 0 | N.A. | 0 | N.A. | 3 |
| Cao 2020 | 1 | 1 | 0 | 1 | 0 | 1 | 0 | 0 | N.A. | 0 | N.A. | 4 |
| Chen 2020 | 1 | 0 | 0 | 1 | 0 | 0 | 0 | 0 | N.A. | 1 | N.A. | 3 |
| Deng 2020a | 1 | 1 | 0 | 0 | 0 | 0 | 0 | 1 | N.A. | 0 | N.A. | 3 |
| Deng 2020b | 1 | 0 | 1 | 0 | 0 | 0 | 0 | 0 | N.A. | 1 | N.A. | 3 |
| Du 2020 | 1 | 0 | 1 | 1 | 0 | 1 | 0 | 0 | N.A. | 1 | N.A. | 5 |
| Guo 2020 | 1 | 1 | 1 | 0 | 0 | 0 | 0 | 0 | N.A. | 0 | N.A. | 3 |
| Huang 2020a | 1 | 1 | 1 | 1 | 0 | 1 | 0 | 0 | N.A. | 1 | N.A. | 6 |
| Huang 2020b | 1 | 0 | 1 | 0 | 0 | 1 | 1 | 0 | N.A. | 0 | N.A. | 4 |
| Huangfu 2020 | 1 | 1 | 0 | 0 | 0 | 0 | 0 | 0 | N.A. | 1 | N.A. | 3 |
| Jiang 2020 | 1 | 1 | 1 | 0 | 0 | 0 | 1 | 0 | N.A. | 1 | N.A. | 5 |
| Lai 2020 | 1 | 1 | 1 | 1 | 0 | 1 | 0 | 0 | N.A. | 1 | N.A. | 6 |
| Li 2020a | 1 | 1 | 1 | 0 | 0 | 1 | 1 | 0 | 1 | N.A. | N.A. | 6 |
| Li 2020b | 1 | 1 | 1 | 0 | 0 | 0 | 1 | 0 | 1 | 0 | N.A. | 5 |
| Li 2020c | 1 | 1 | 0 | 1 | 0 | 1 | 0 | 0 | N.A. | 1 | N.A. | 5 |
| Li 2020d | 1 | 1 | 1 | 1 | 0 | 1 | 0 | 0 | N.A. | 1 | N.A. | 6 |
| Li 2020e | 1 | 0 | 1 | 0 | 0 | 1 | 0 | 0 | N.A. | 0 | N.A. | 3 |
| Liang 2020 | 1 | 0 | 1 | 0 | 0 | 0 | 0 | 0 | N.A. | 0 | N.A. | 2 |
| Liu 2020a | 1 | 0 | 1 | 0 | 0 | 1 | 0 | 0 | N.A. | 0 | N.A. | 3 |
| Liu 2020b | 1 | 0 | 1 | 0 | 0 | 1 | 0 | 0 | N.A. | 1 | N.A. | 4 |
| Liu 2020c | 1 | 1 | 1 | 1 | 0 | 0 | 0 | 0 | N.A. | 0 | N.A. | 4 |
| Luo 2020 | 1 | 1 | 1 | 1 | 0 | 0 | 0 | 1 | N.A. | 1 | N.A. | 6 |
| Lv 2020 | 1 | 1 | 0 | 0 | 0 | 0 | 1 | 0 | N.A. | 0 | N.A. | 3 |
| Mo 2020 | 1 | 1 | 1 | 0 | 0 | 1 | 0 | 0 | N.A. | 1 | N.A. | 5 |
| Pu 2020 | 1 | 1 | 0 | 1 | 0 | 0 | 0 | 0 | N.A. | 0 | N.A. | 3 |
| Qi 2020 | 1 | 1 | 1 | 0 | 0 | 0 | 1 | 1 | 1 | 1 | N.A. | 7 |
| Sheng 2020 | 1 | 1 | 0 | 1 | 0 | 1 | 0 | 0 | N.A. | 0 | N.A. | 4 |
| Song 2020 | 1 | 0 | 1 | 0 | 0 | 1 | 0 | 0 | N.A. | 0 | N.A. | 3 |
| Sun 2020 | 1 | 1 | 1 | 0 | 0 | 1 | 1 | 0 | N.A. | 0 | N.A. | 5 |
| Tian 2020 | 1 | 0 | 1 | 1 | 0 | 0 | 0 | 0 | N.A. | 1 | N.A. | 4 |
| Wang 2020 | 1 | 1 | 0 | 0 | 0 | 0 | 0 | 0 | N.A. | 0 | N.A. | 2 |
| Wu 2020a | 1 | 1 | 0 | 0 | 0 | 1 | 0 | 0 | N.A. | 1 | N.A. | 4 |
| Wu 2020b | 1 | 1 | 0 | 0 | 0 | 1 | 0 | 0 | N.A. | 0 | N.A. | 3 |
| Xiao 2020a | 1 | 0 | 1 | 0 | 0 | 1 | 0 | 0 | N.A. | 1 | N.A. | 4 |
| Xiao 2020b | 1 | 0 | 1 | 0 | 0 | 0 | 0 | 0 | N.A. | 0 | N.A. | 2 |
| Xing 2020 | 1 | 1 | 1 | 1 | 0 | 0 | 0 | 0 | N.A. | 0 | N.A. | 4 |
| Xu 2020a | 1 | 1 | 0 | 1 | 0 | 0 | 0 | 0 | N.A. | 1 | N.A. | 4 |
| Xu 2020b | 1 | 1 | 1 | 1 | 0 | 0 | 0 | 0 | N.A. | 1 | N.A. | 5 |
| Yin 2020 | 1 | 1 | 1 | 1 | 0 | 1 | 1 | 1 | N.A. | 1 | N.A. | 8 |
| Yuan 2020 | 1 | 1 | 1 | 0 | 0 | 0 | 0 | 0 | N.A. | 1 | N.A. | 4 |
| Zhang 2020a | 1 | 0 | 1 | 0 | 0 | 1 | 0 | 0 | N.A. | 0 | N.A. | 3 |
| Zhang 2020b | 1 | 1 | 1 | 0 | 0 | 1 | 1 | 0 | N.A. | 0 | N.A. | 5 |
| Zhu 2020a | 1 | 1 | 1 | 0 | 0 | 1 | 0 | 1 | N.A. | 1 | N.A. | 6 |
| Zhu 2020b | 1 | 1 | 1 | 1 | 0 | 1 | 1 | 0 | 1 | 1 | N.A. | 8 |

Note. Abbreviations: AHRQ = Agency for Healthcare Research and Quality; N.A. not applicable. The total score is added up from all applicable items.

Item 1: Define the source of information (survey, record review). To be rated as 1, the study should provide an appropriate description of the source of the survey.

Item 2: List inclusion and exclusion criteria for exposed and unexposed subjects (cases and controls) or refer to previous publications. To be rated as 1, the study should report both the inclusion and exclusion criteria of participants. Studies with only the description of inclusion criteria were rated as 0 on this item.

Item 3: Indicate time period used for identifying patients. Studies without a clear time period of the survey were rated as 0 on this item.

Item 4: Indicate whether or not subjects were consecutive if not population-based. To be as rated 1, the study should provide clear information to prove that the sample could represent the target population. Studies with convenient samples were rated 0 on this item.

Item 5: Indicate if evaluators of subjective components of study were masked to other aspects of the status of the participants. Since all included studies only used self-rated questionnaires, and all participants were aware of their exposure, mask could not be achieved. All studies were rated as 0 on this item.

Item 6: Describe any assessments undertaken for quality assurance purposes (e.g. test/retest of outcome measurements). To be rated as 1, the study need to report the reliability and validity of questionnaires used.

Item 7: Explain any patient exclusions from analysis. To be rated as 1, the study should report that all patients were included in the analysis, or explain why any patient was excluded.

Item 8: Describe how confounding was assessed and/or control. The previous history of mental illness should be one of the important confounding factors in these studies. Therefore, studies with inadequate information about how confounding was assessed or controlled were rated as 0 on this item.

Item 9: If applicable, explain how missing data were handled explained in the analysis. To be rated as 1, the study should report that how missing data was handled. If there’s no information about whether there’s missing data or not, the study would be rated as not applicable (N.A.) on this item.

Item 10: Summarize patient response rates and completeness of data collection. To be rated as 1, the study should report the response rate and completeness of data collection. For studies with convenient sampling, no response rate could be estimated; therefore, they were rated as 0 on this item.

Item 11: Clarify what follow-up, if any, was expected and the percentage of patients for which incomplete data or follow-up was obtained. Since all included studies did not report any plan of follow-up or repeated measurements, they were rated as N.A. on this item.


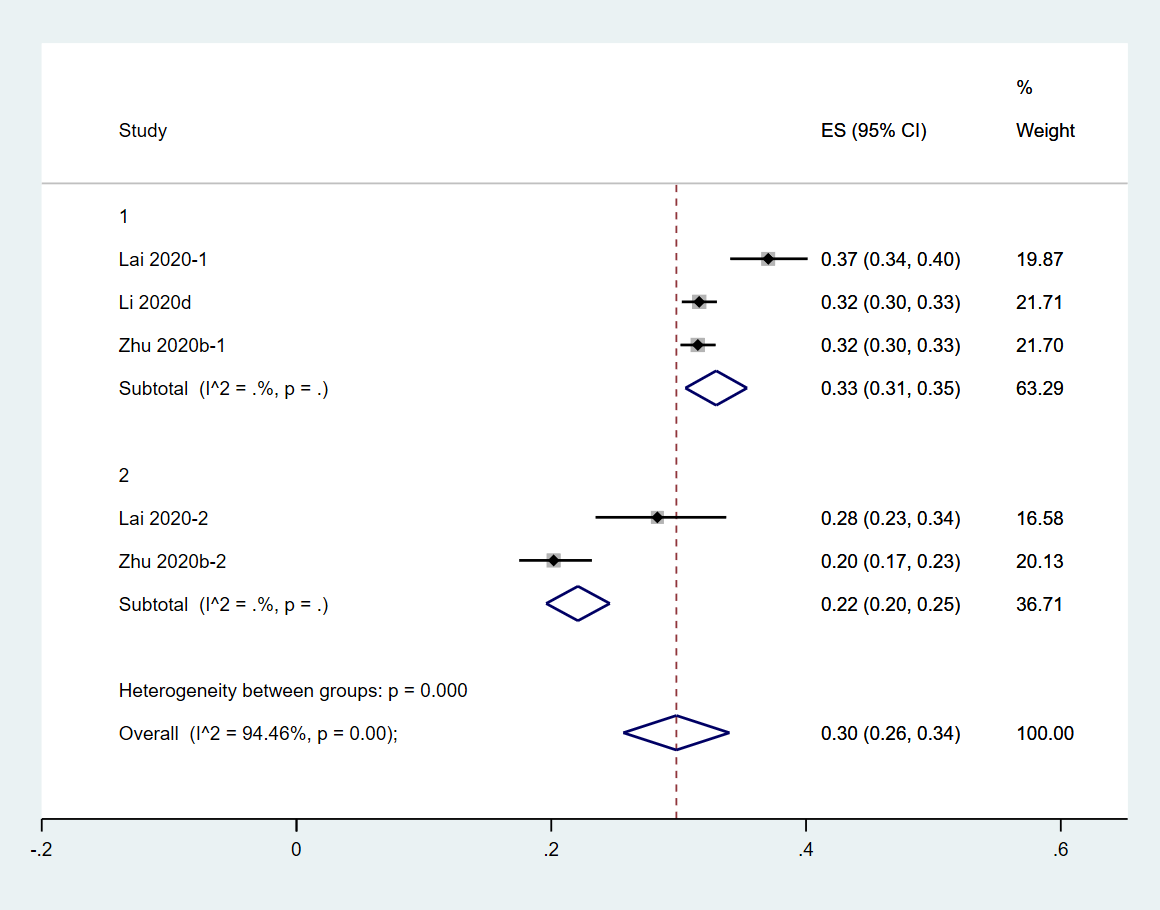


S. Fig.1 the prevalence of moderate to severe level of PTSS in female vs. male HCW


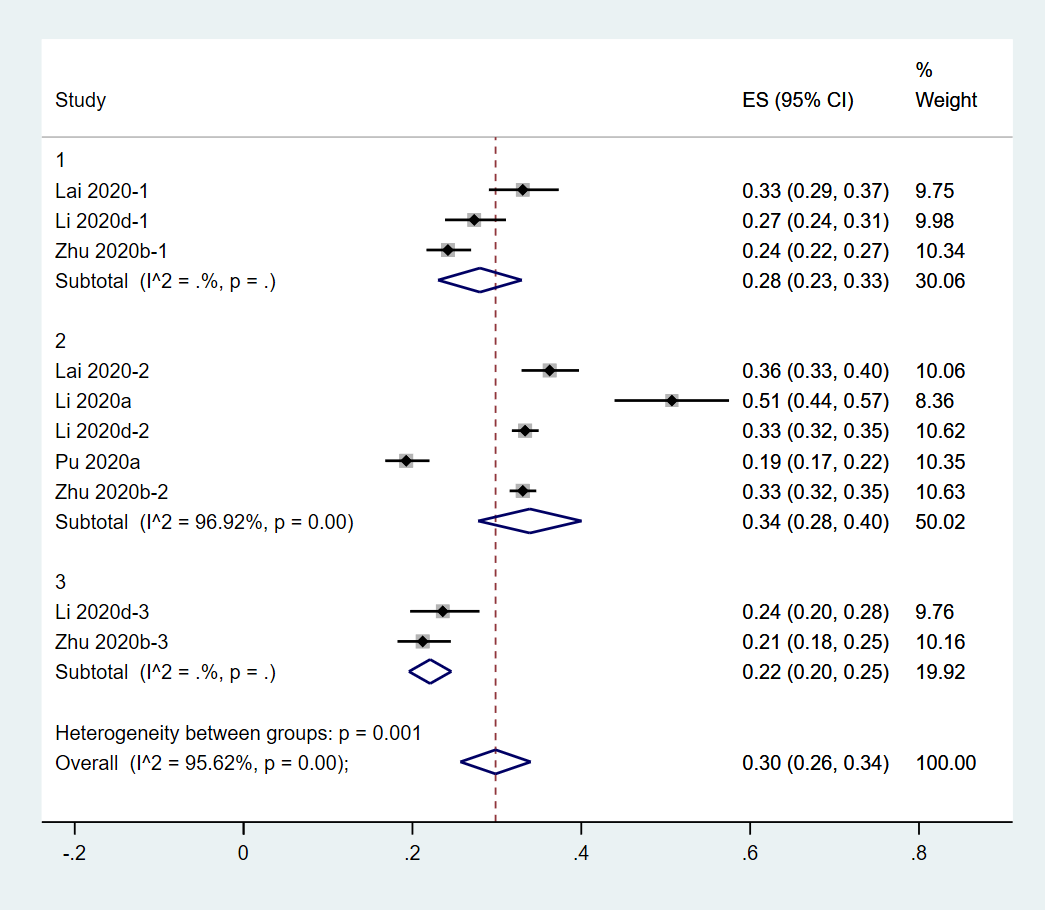


S. Fig.2 the prevalence of moderate to severe level of PTSS in doctors vs. nurses vs. technicians


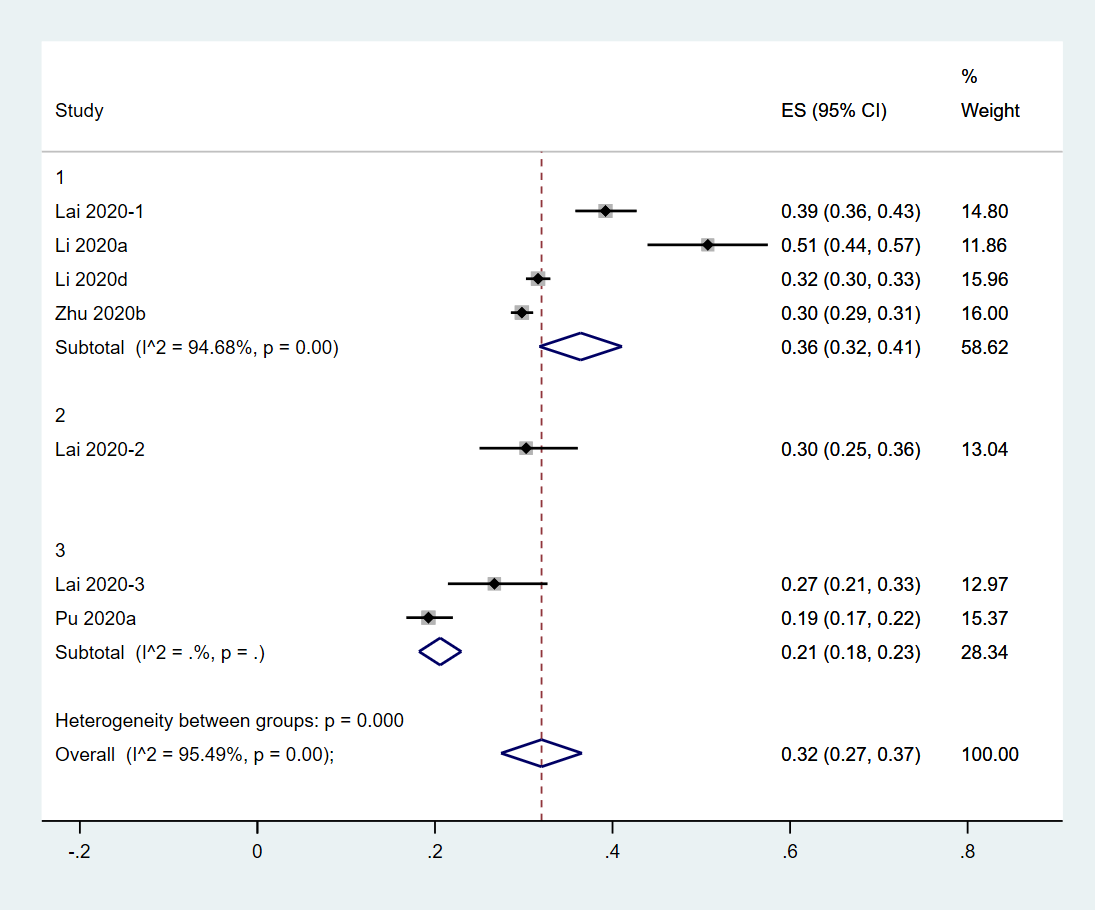


S. Fig.3 the prevalence of moderate to severe level of PTSS in HCW from Wuhan vs. other cities in the Hubei province vs. other provinces in China


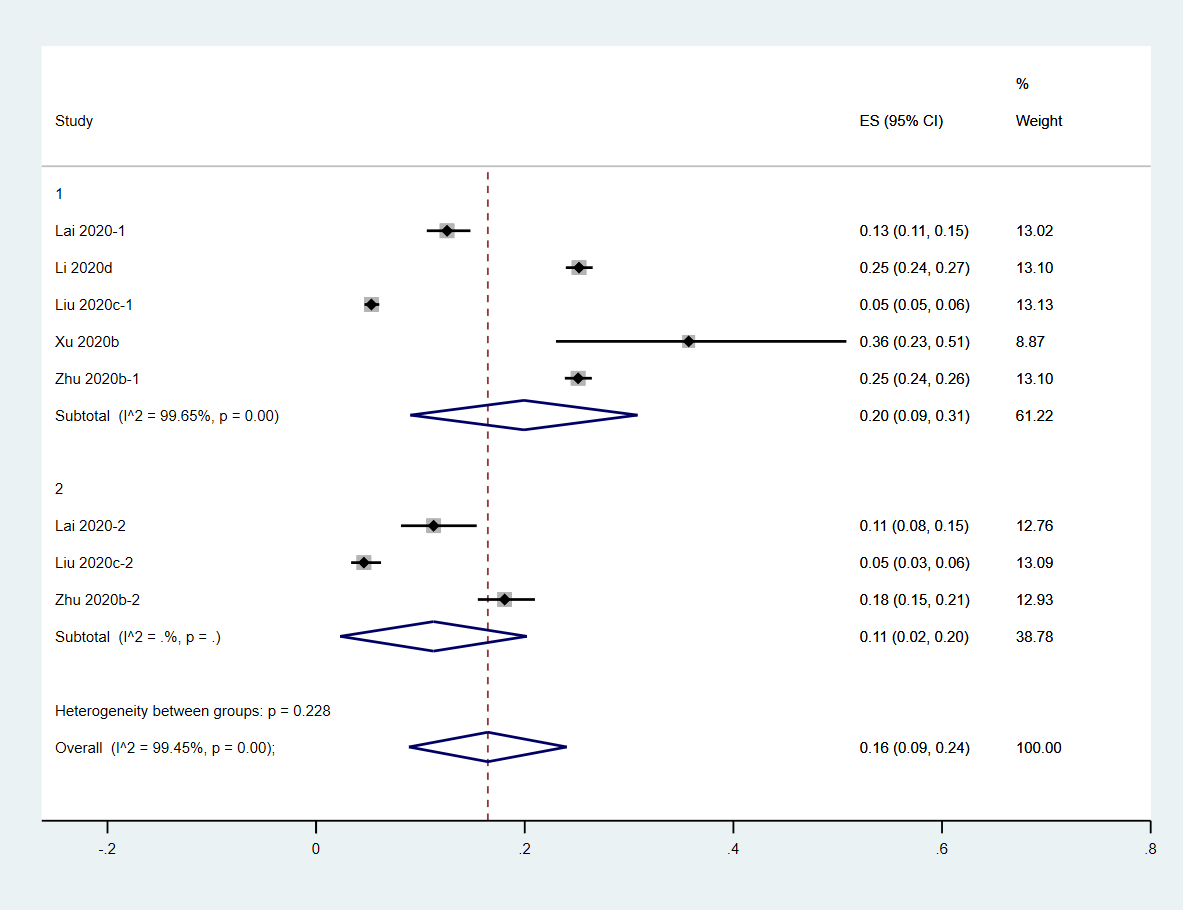


S. Fig.4 the prevalence of moderate to severe level of anxiety in female vs. male HCW


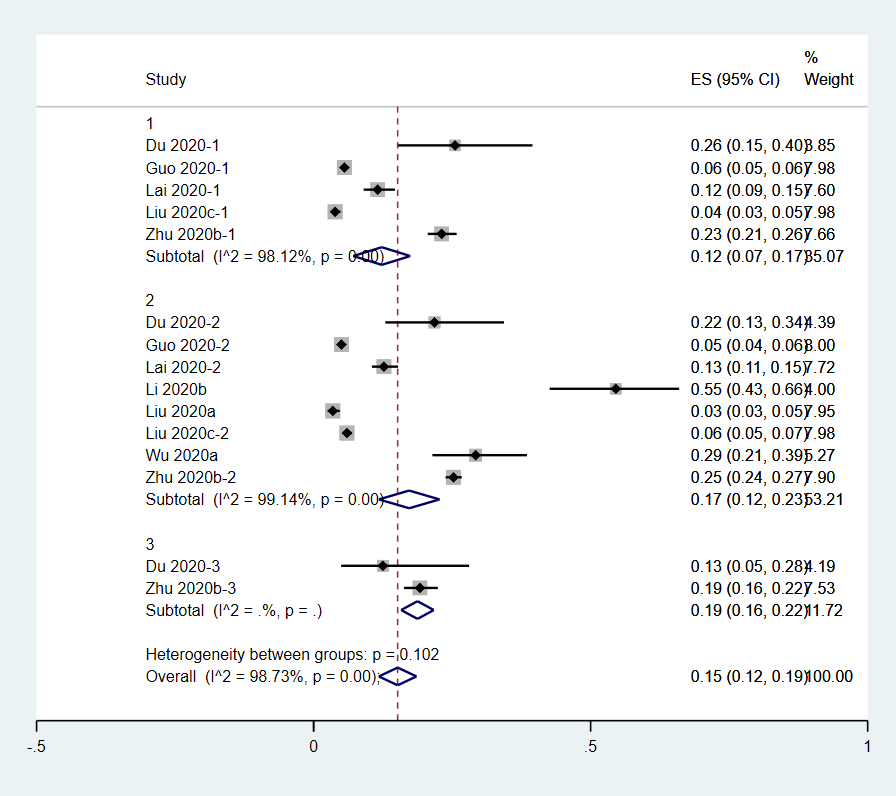


S. Fig.5 the prevalence of moderate to severe level of anxiety in doctors vs. nurses vs. technicians


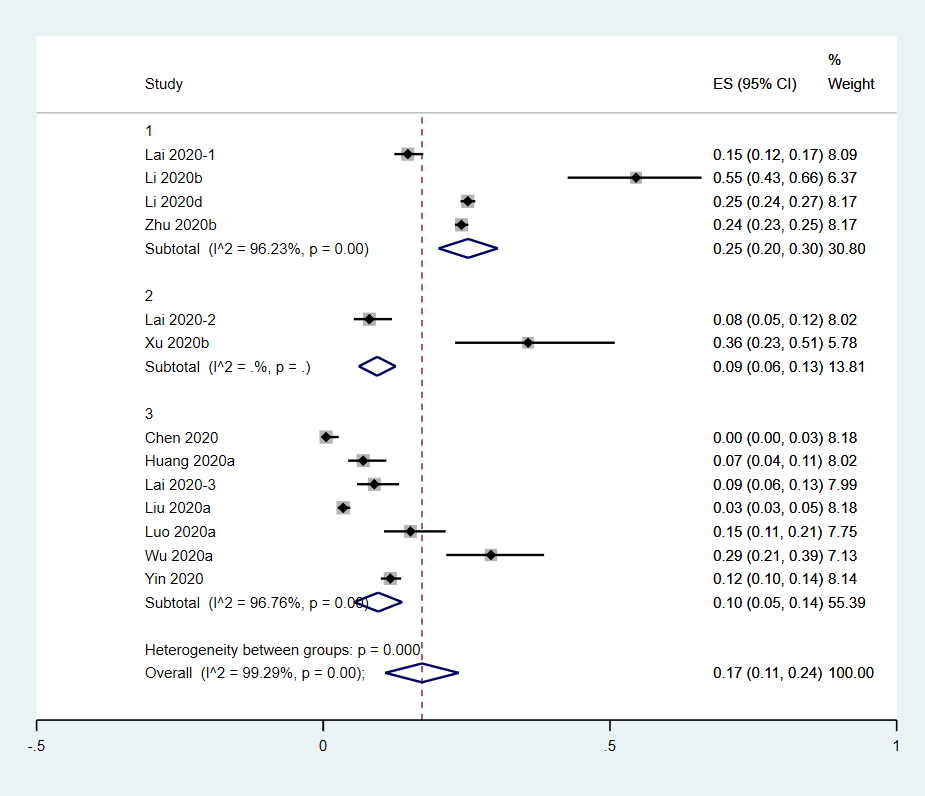


S. Fig.6 the prevalence of moderate to severe level of anxiety in HCW from Wuhan vs. other cities in the Hubei province vs. other provinces in China


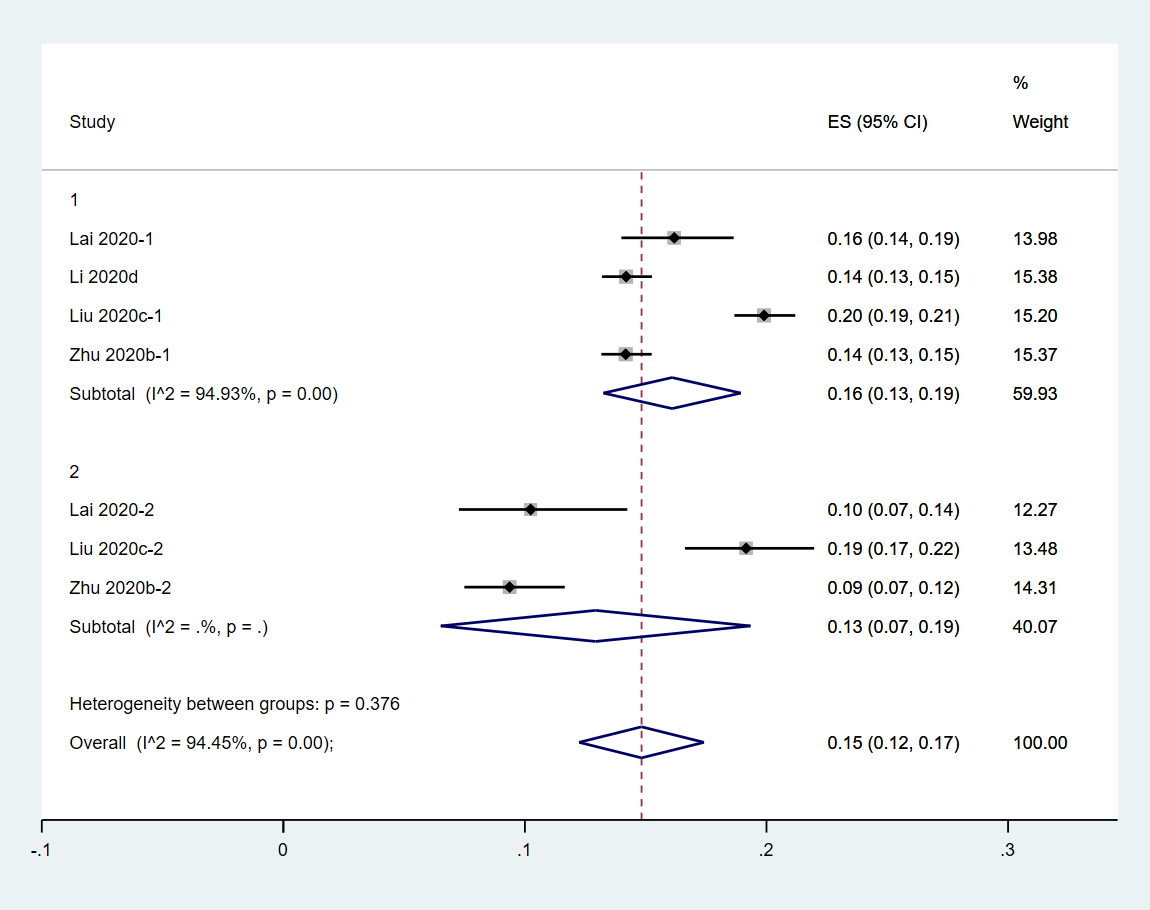


S. Fig.7 the prevalence of moderate to severe level of depression in female vs. male HCW


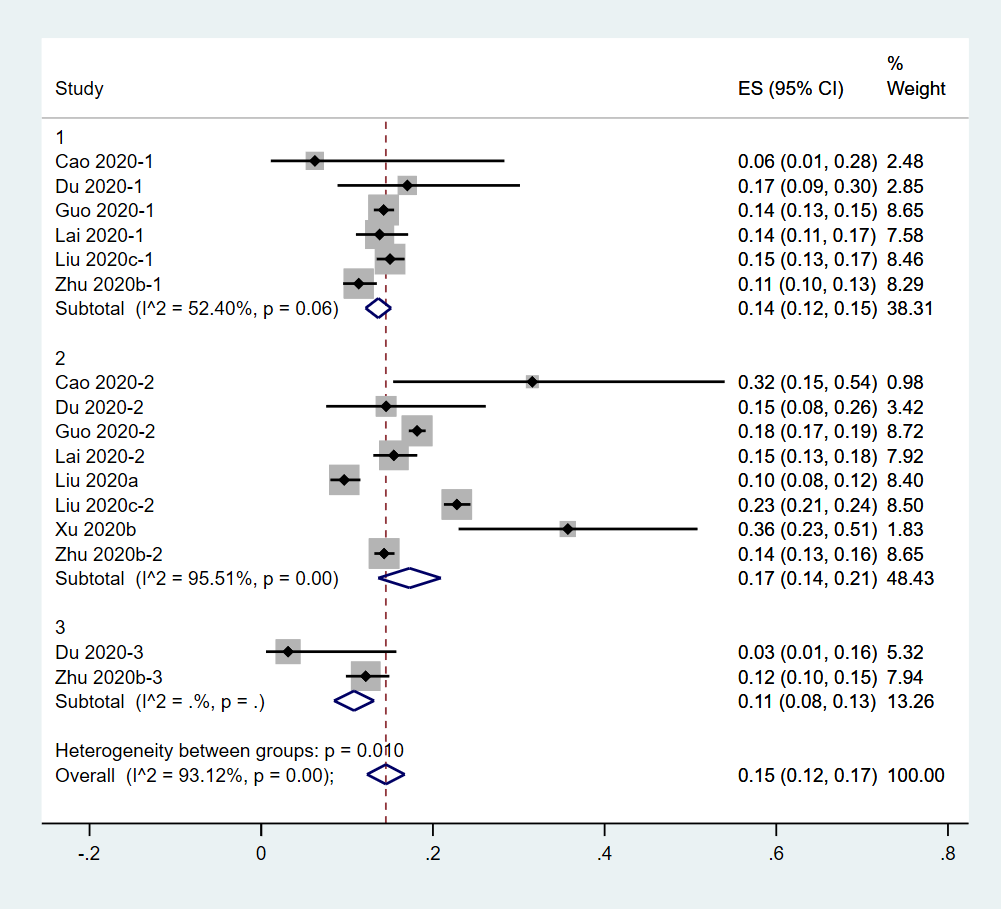


S. Fig.8 the prevalence of moderate to severe level of depression in doctors vs. nurses vs. technicians


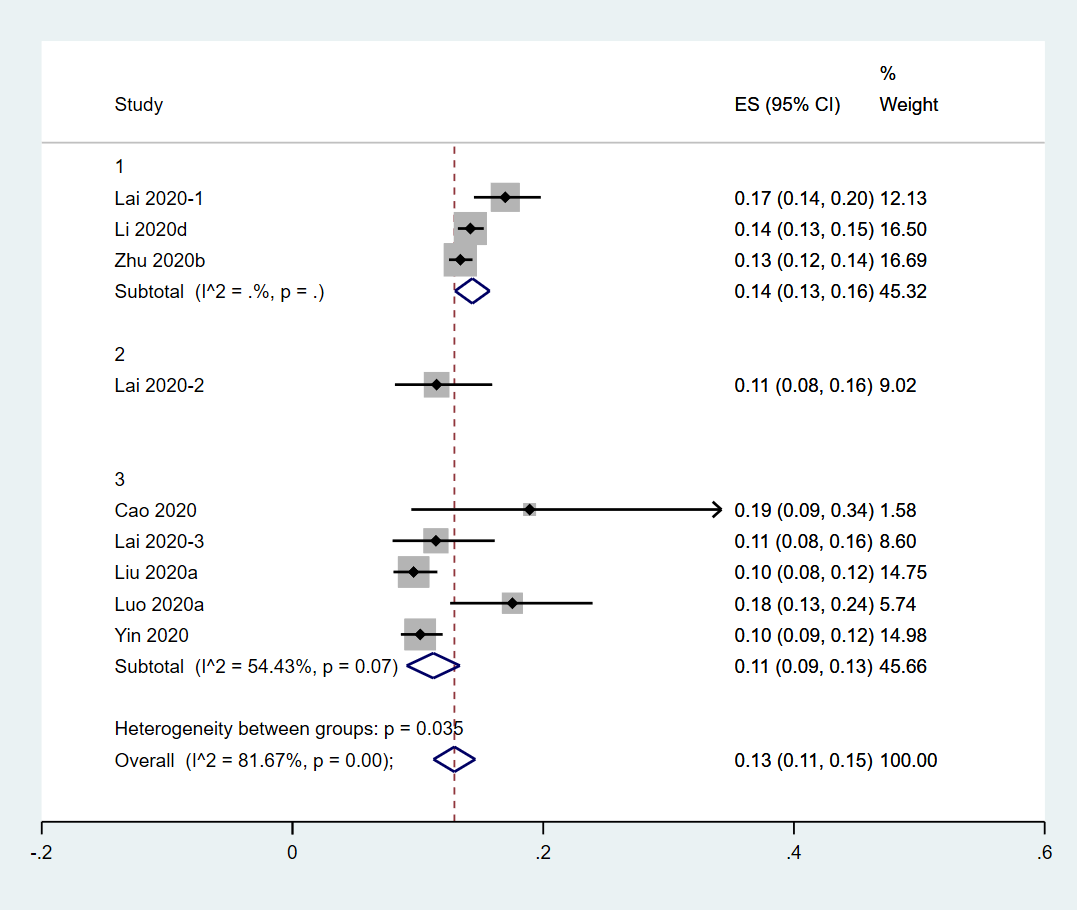


S. Fig.9 the prevalence of moderate to severe level of depression in HCW from Wuhan vs. other cities in the Hubei province vs. other provinces in China


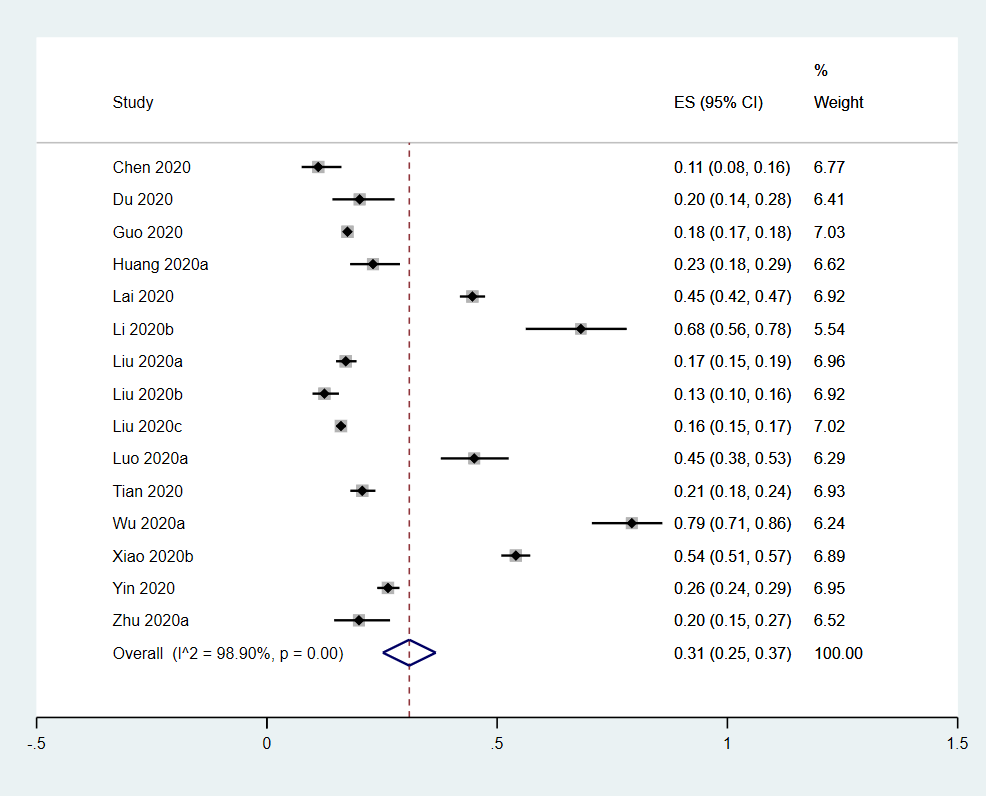


S. Fig.10 the prevalence of mild to severe level of anxiety in the whole sample


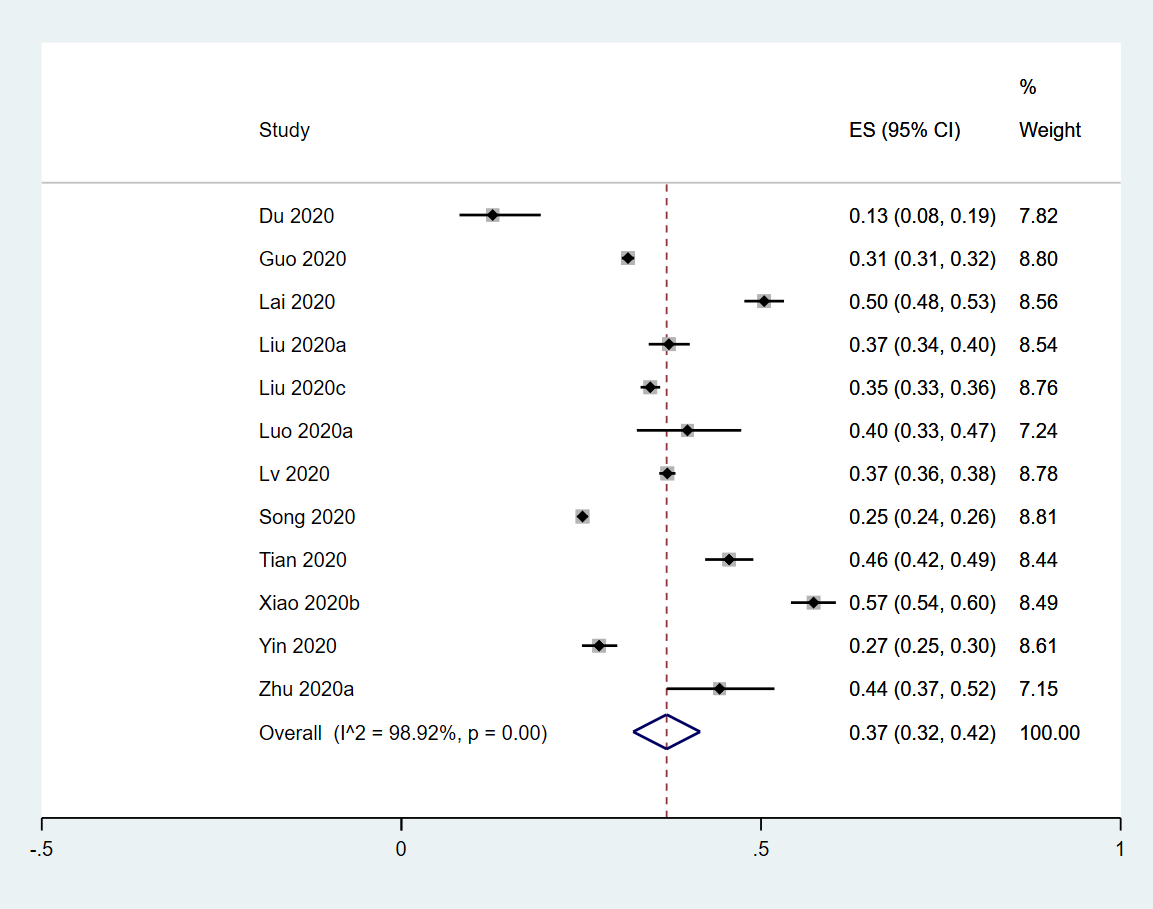


S. Fig.11 the prevalence of mild to severe level of depression in the whole sample


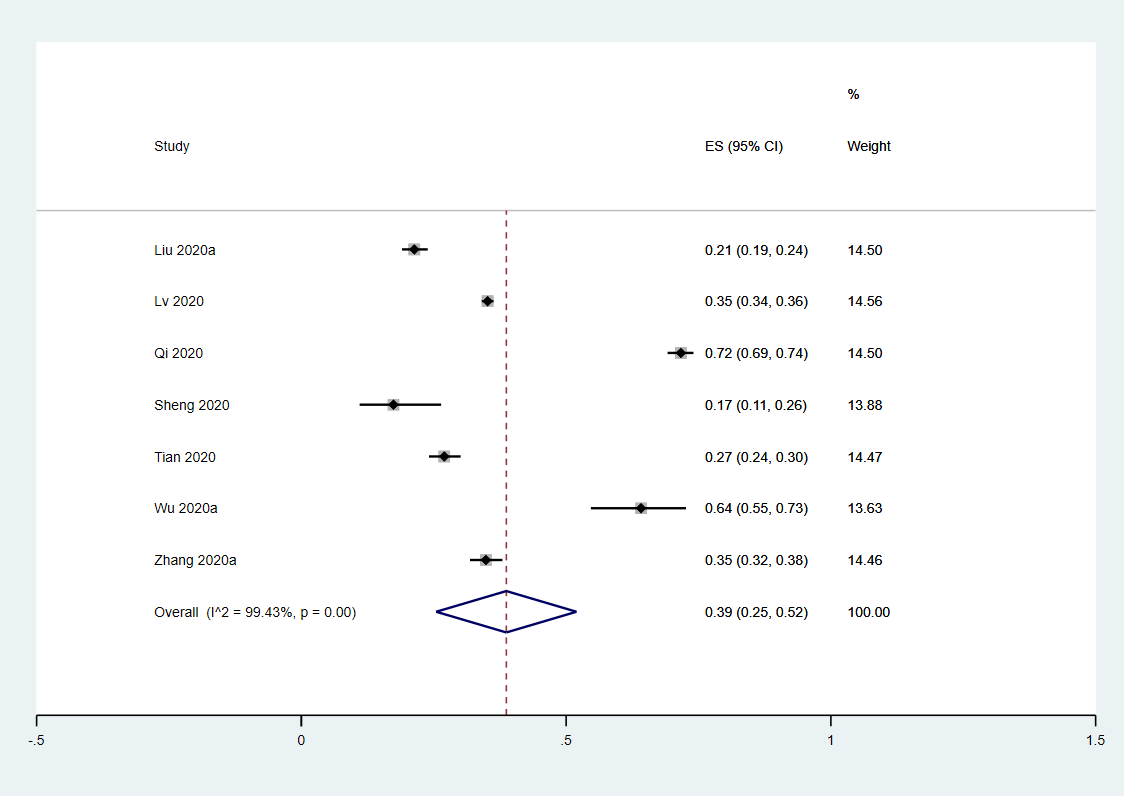


S. Fig.12 the prevalence of mild to severe level of sleep disturbance in the whole sample


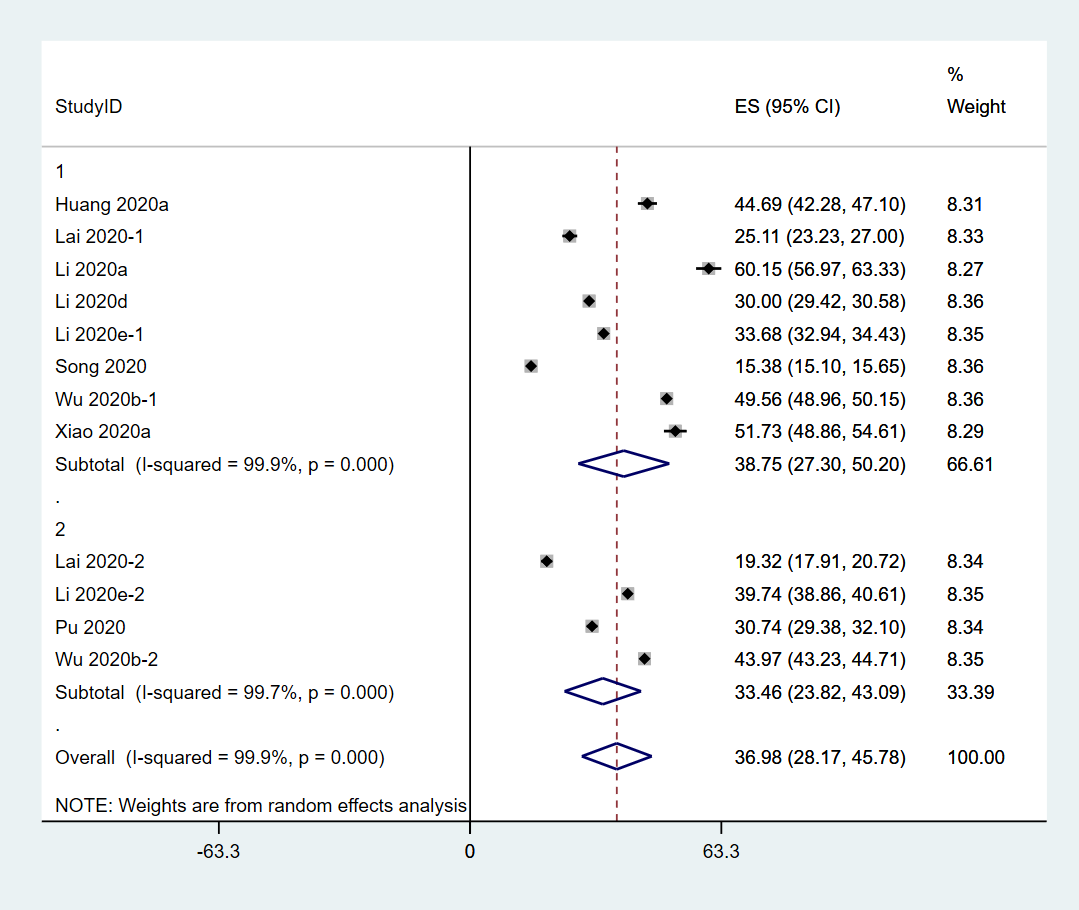


S. Fig.13 the severity of PTSS in frontline vs. non-frontline HCW


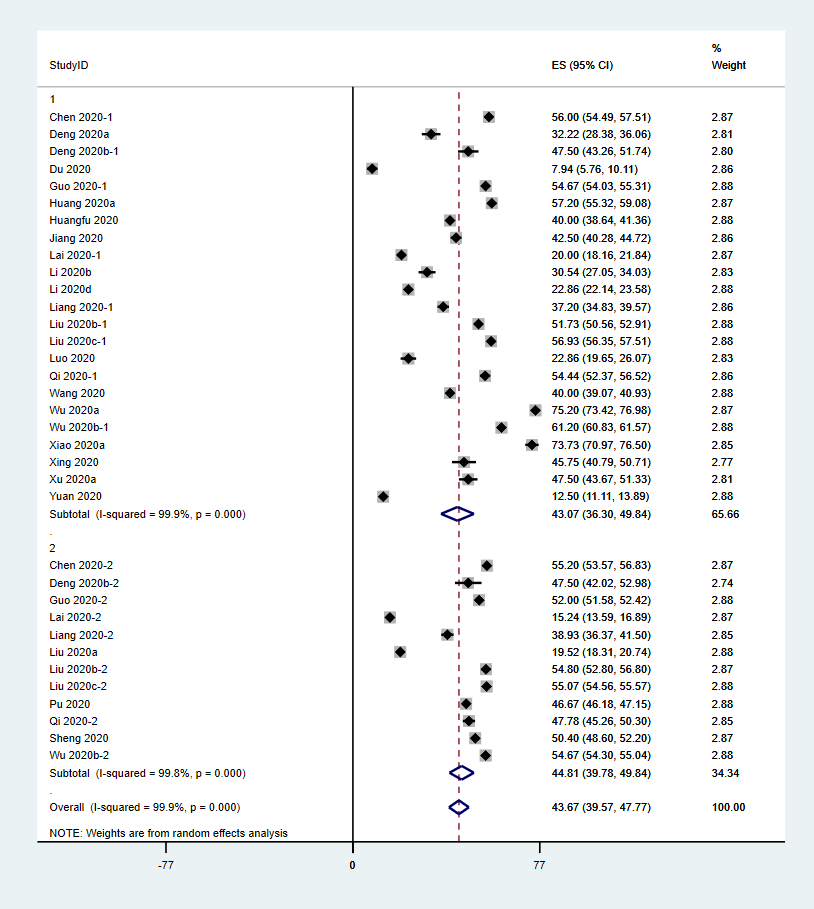


S. Fig.14 the severity of anxiety in frontline vs. non-frontline HCW


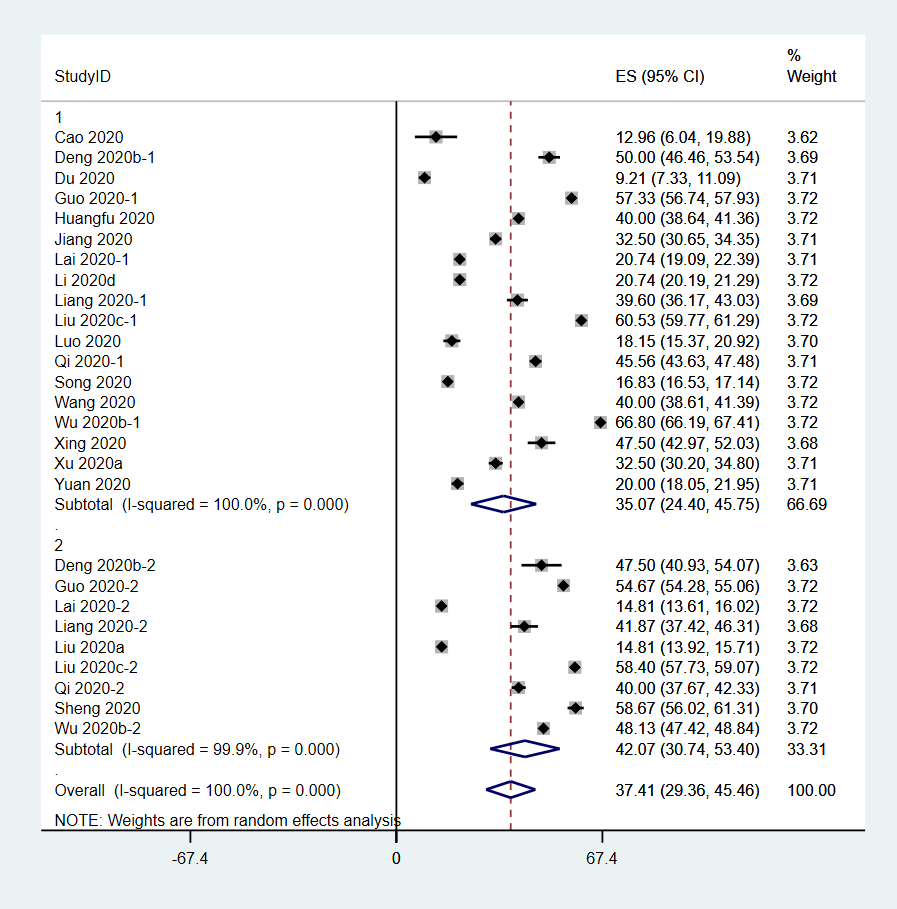


S. Fig.15 the severity of depression in frontline vs. non-frontline HCW


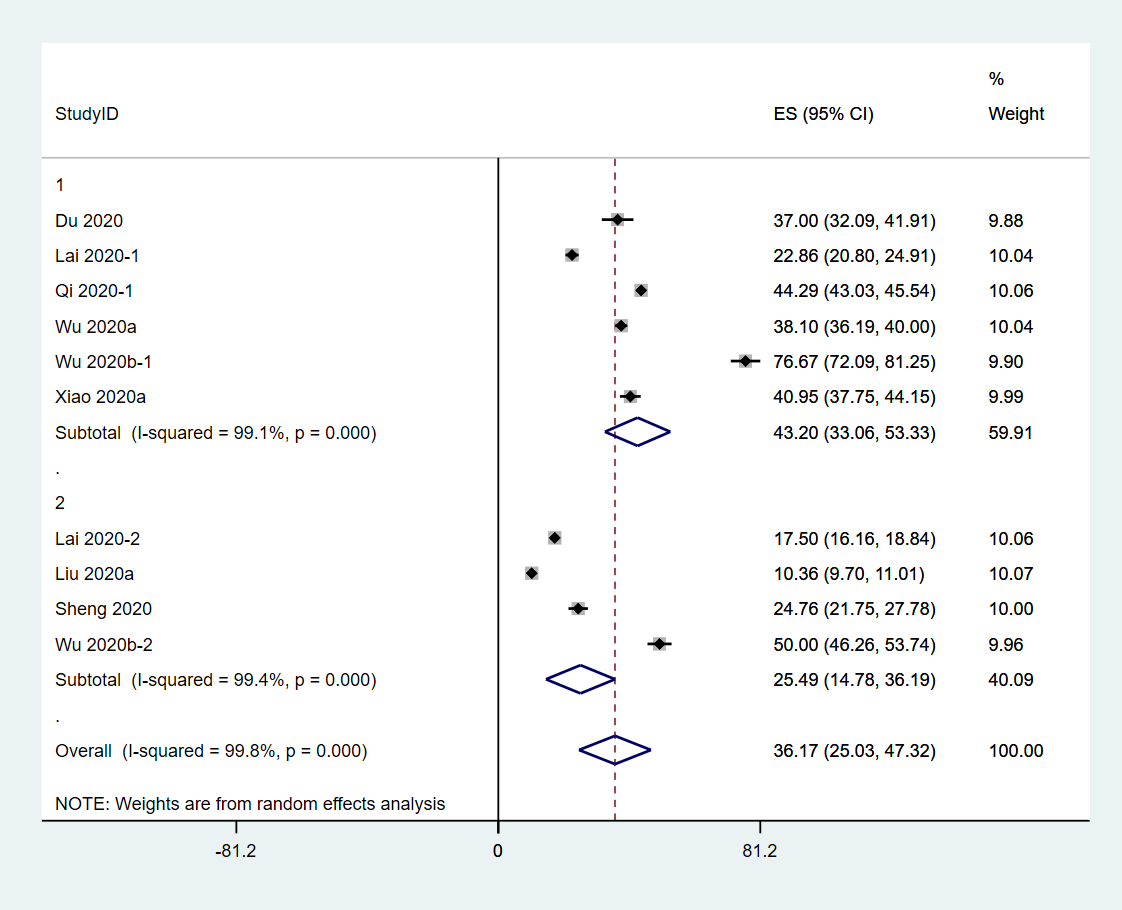


S. Fig.16 the severity of sleep disturbances in frontline vs. non-frontline HCW


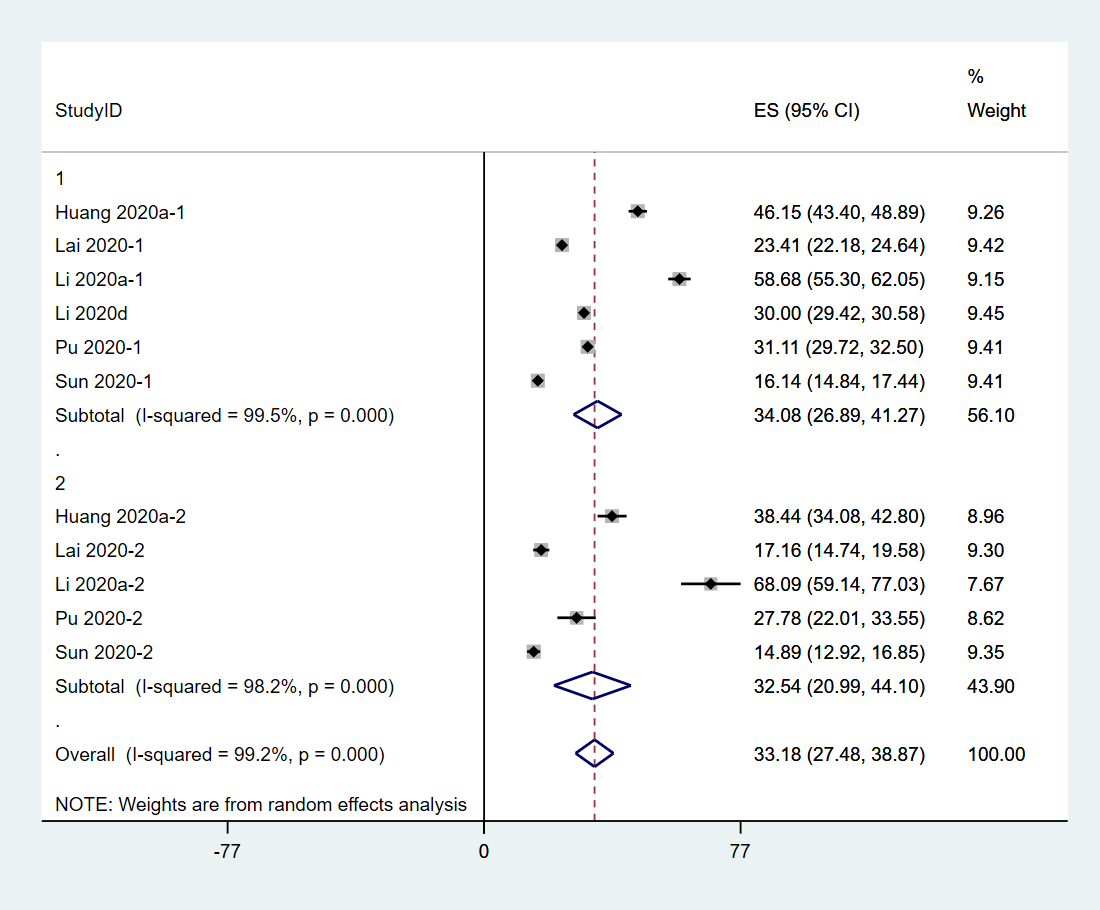


S. Fig.17 the severity of PTSS in female vs. male HCW


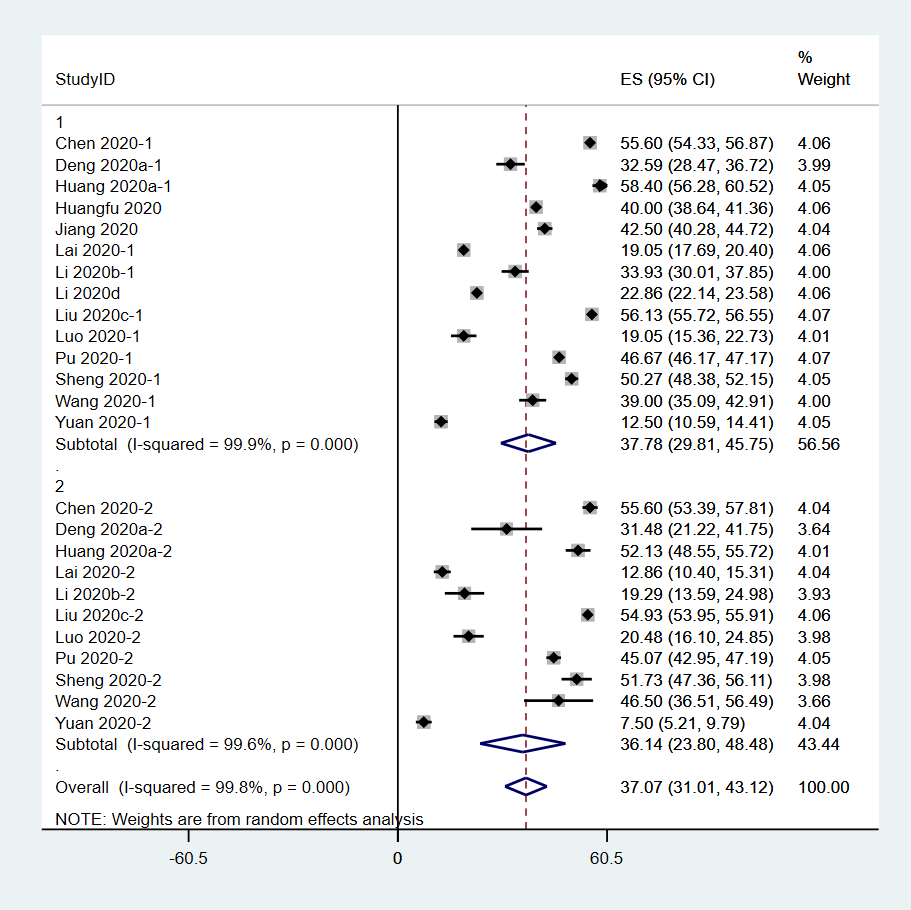


S. Fig.18 the severity of anxiety in female vs. male HCW


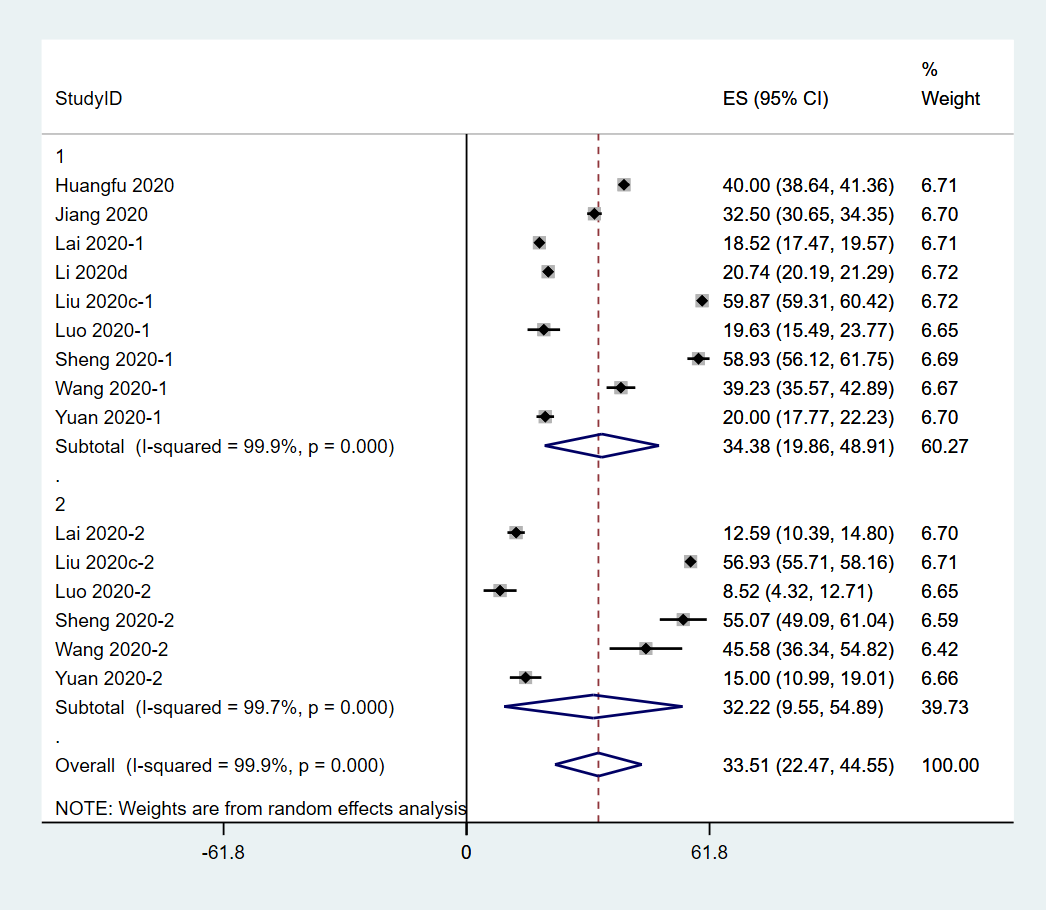


S. Fig.19 the severity of depression in female vs. male HCW


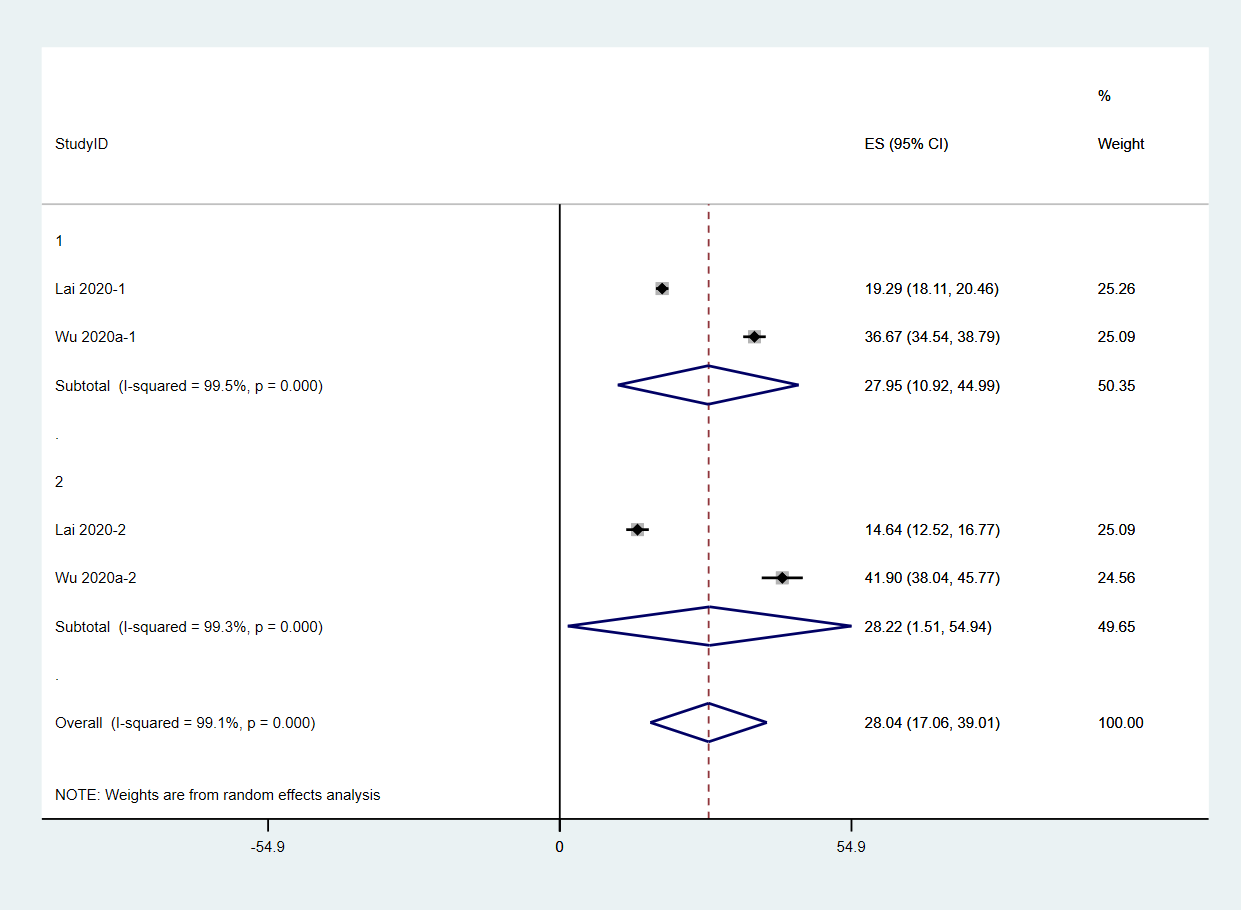


S. Fig.20 the severity of sleep disturbances in female vs. male HCW


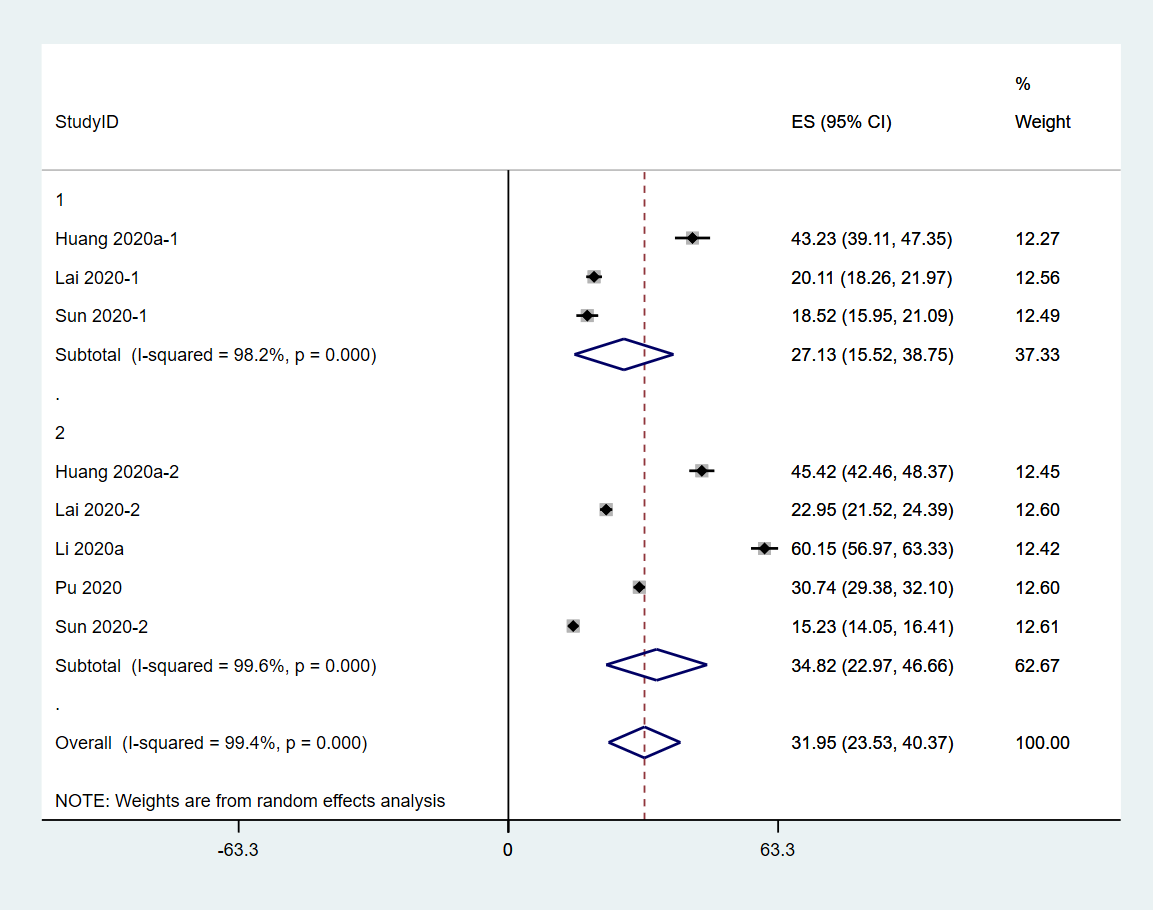


S. Fig.21 the severity of PTSS in doctors vs. nurses


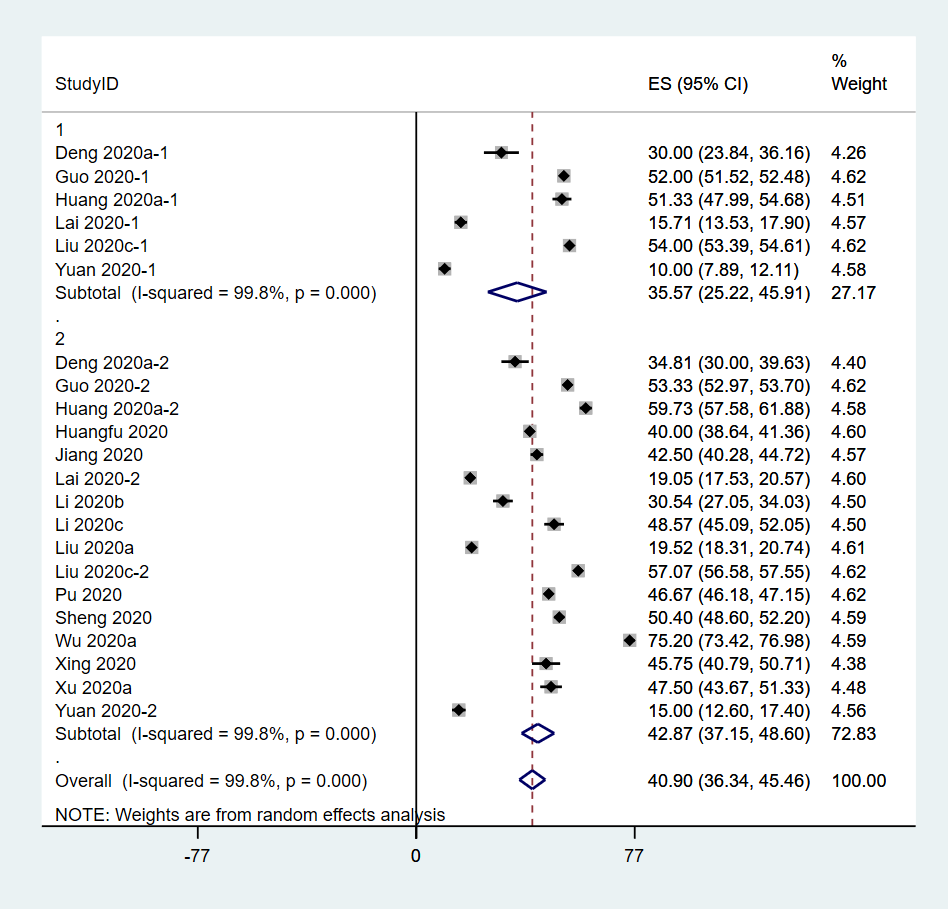


S. Fig.22 the severity of anxiety in doctors vs. nurses


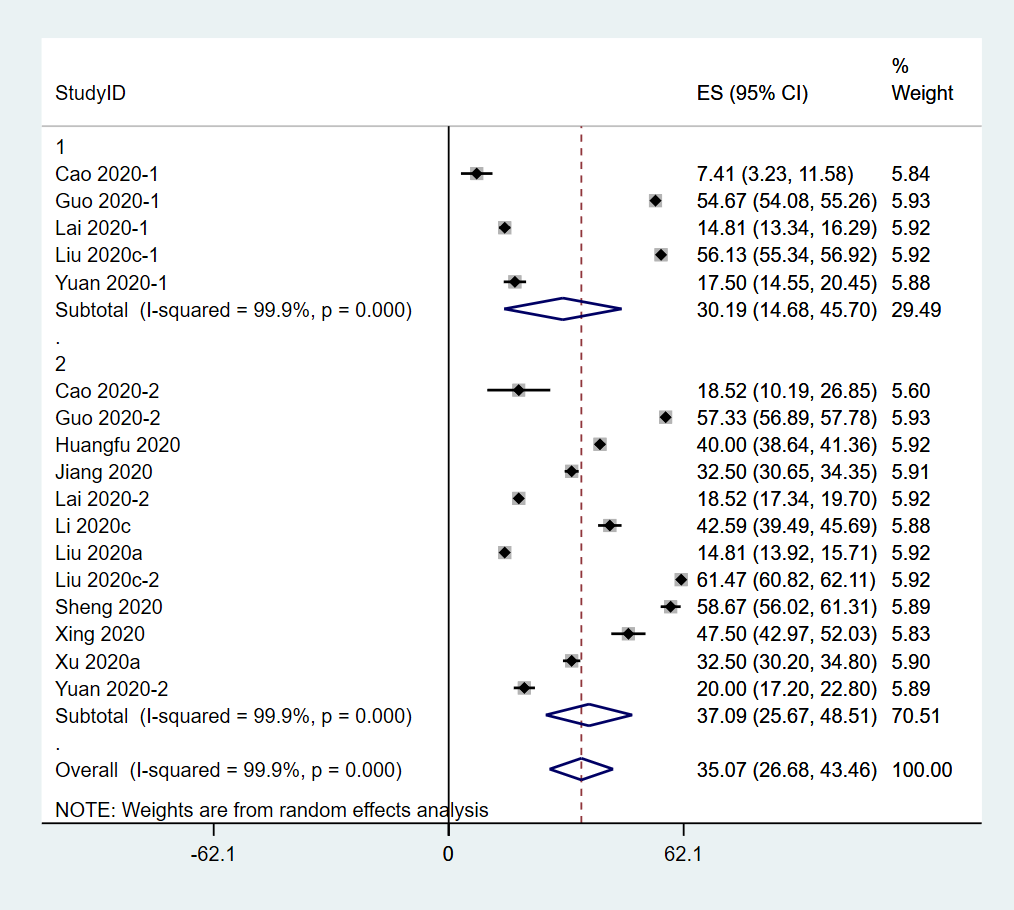


S. Fig.23 the severity of depression in doctors vs. nurses


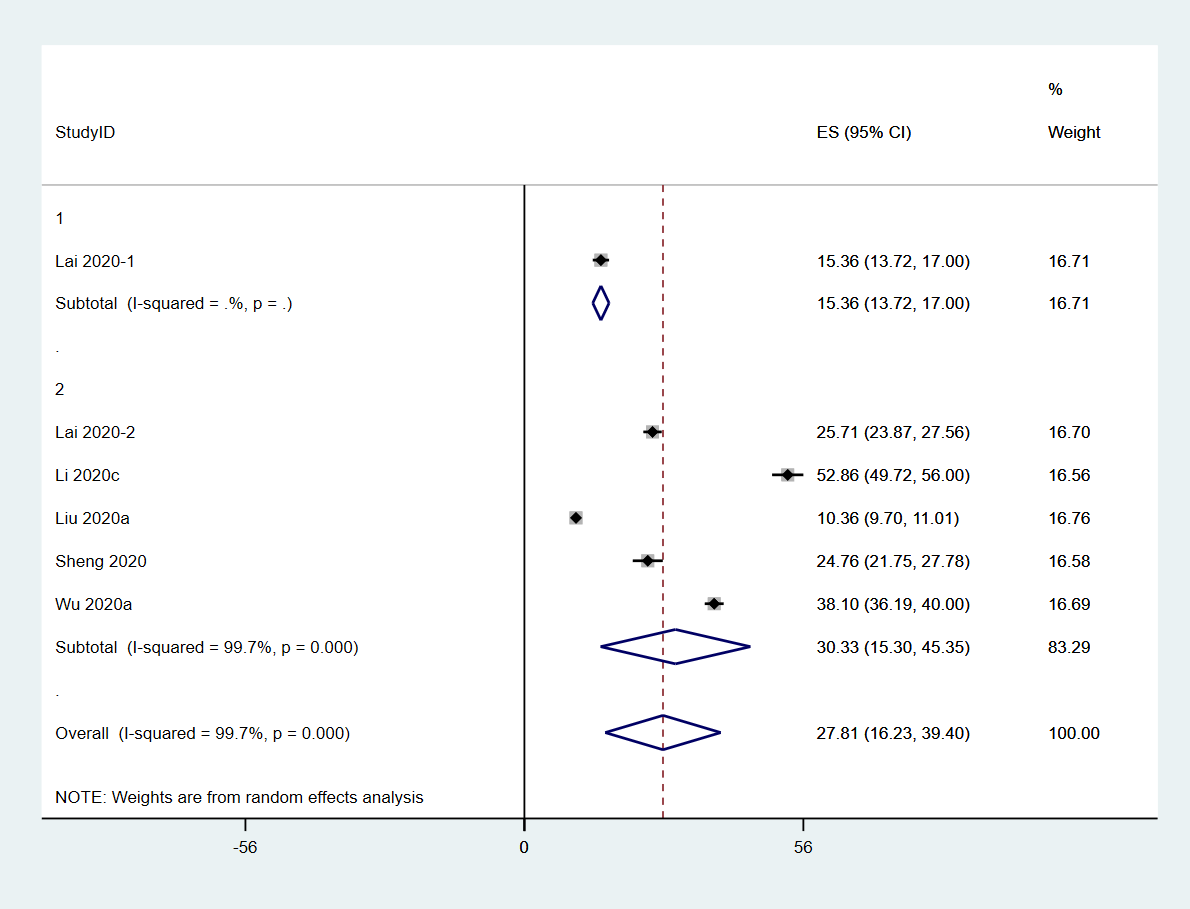


S. Fig.24 the severity of sleep disturbances in doctors vs. nurses


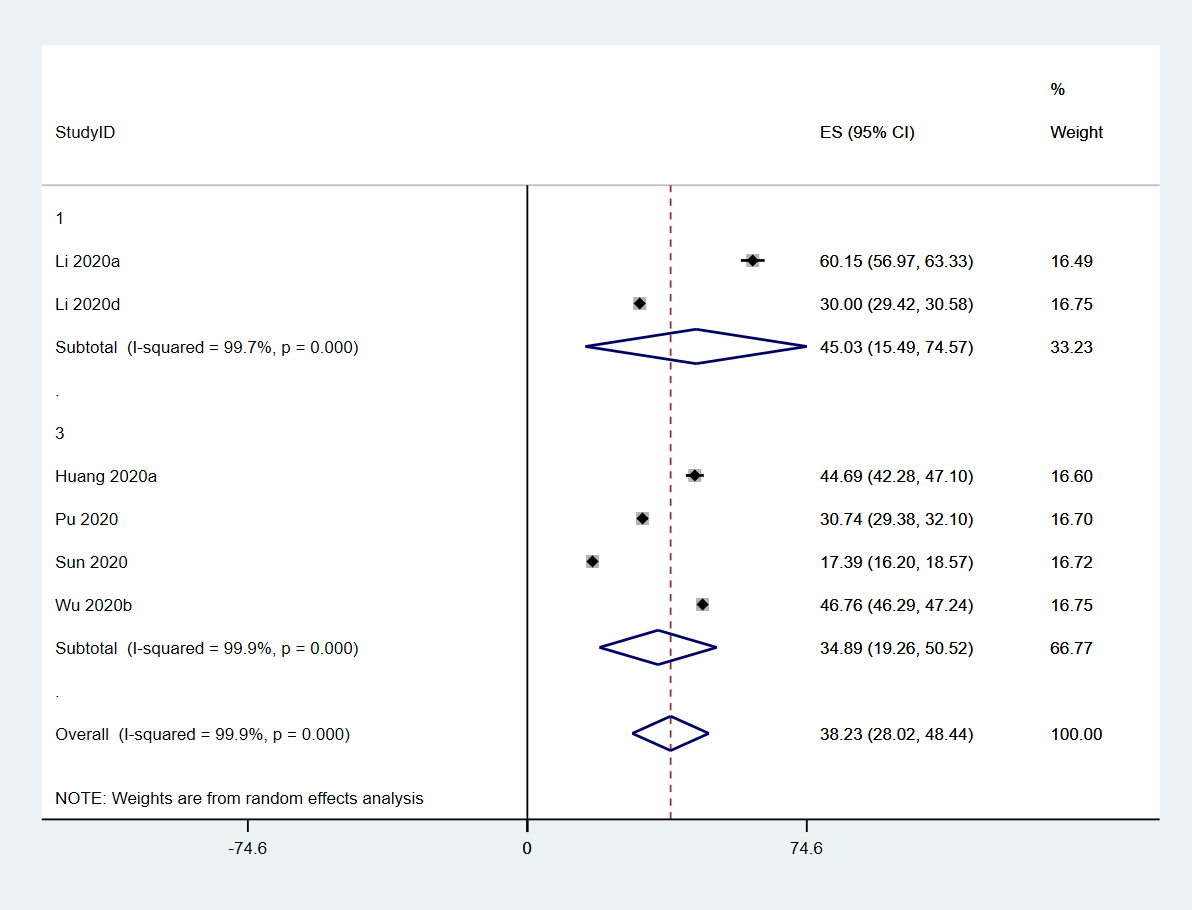


S. Fig.25 the severity of PTSS of HCW in Wuhan vs. other provinces in China


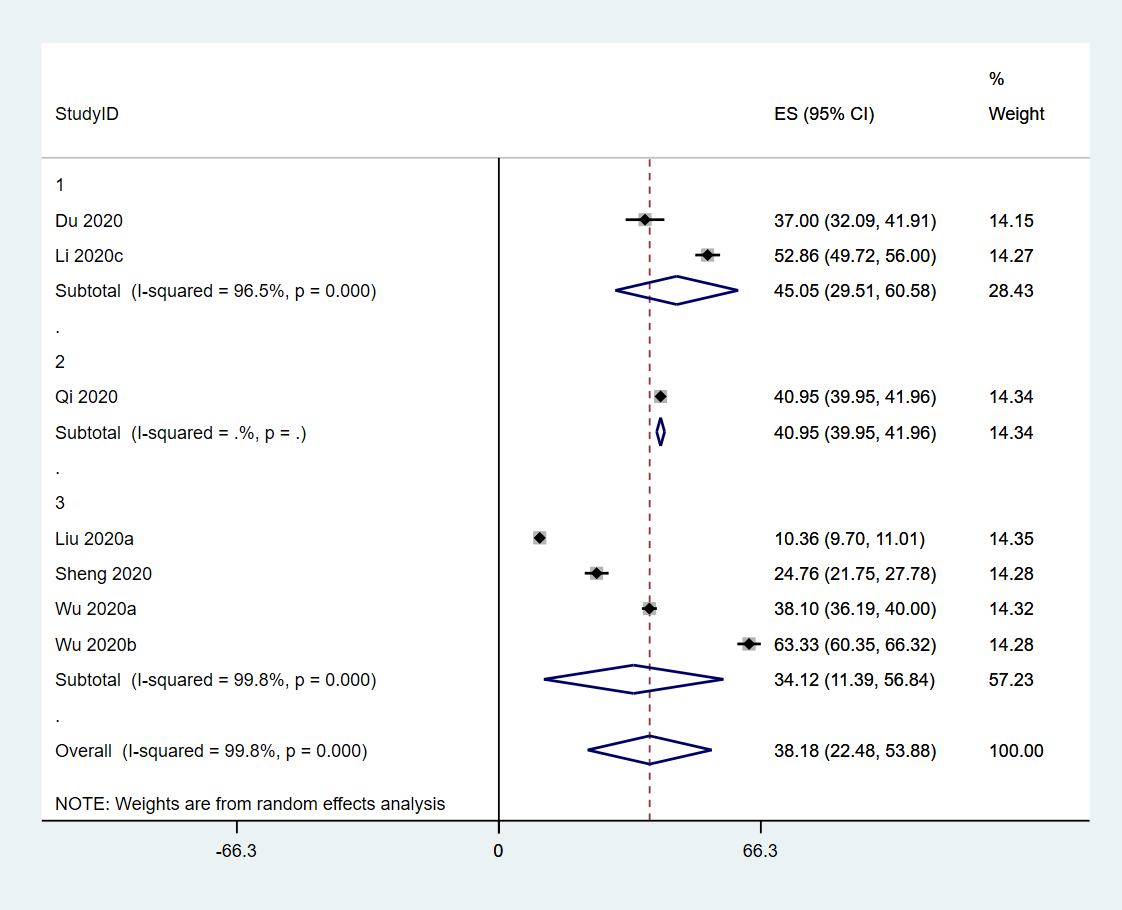


S. Fig.26 the severity of sleep disturbance of HCW in Wuhan vs. other provinces in China


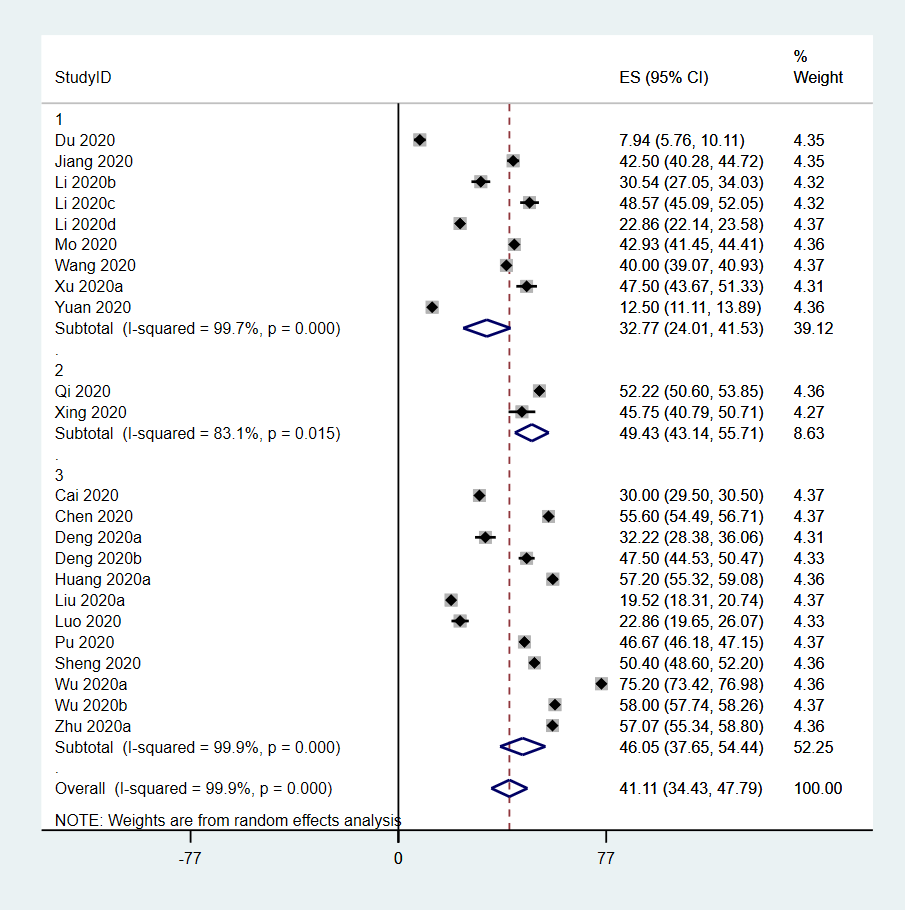


S. Fig.27 the severity of anxiety of HCW in Wuhan vs. Hubei province vs. other provinces in China


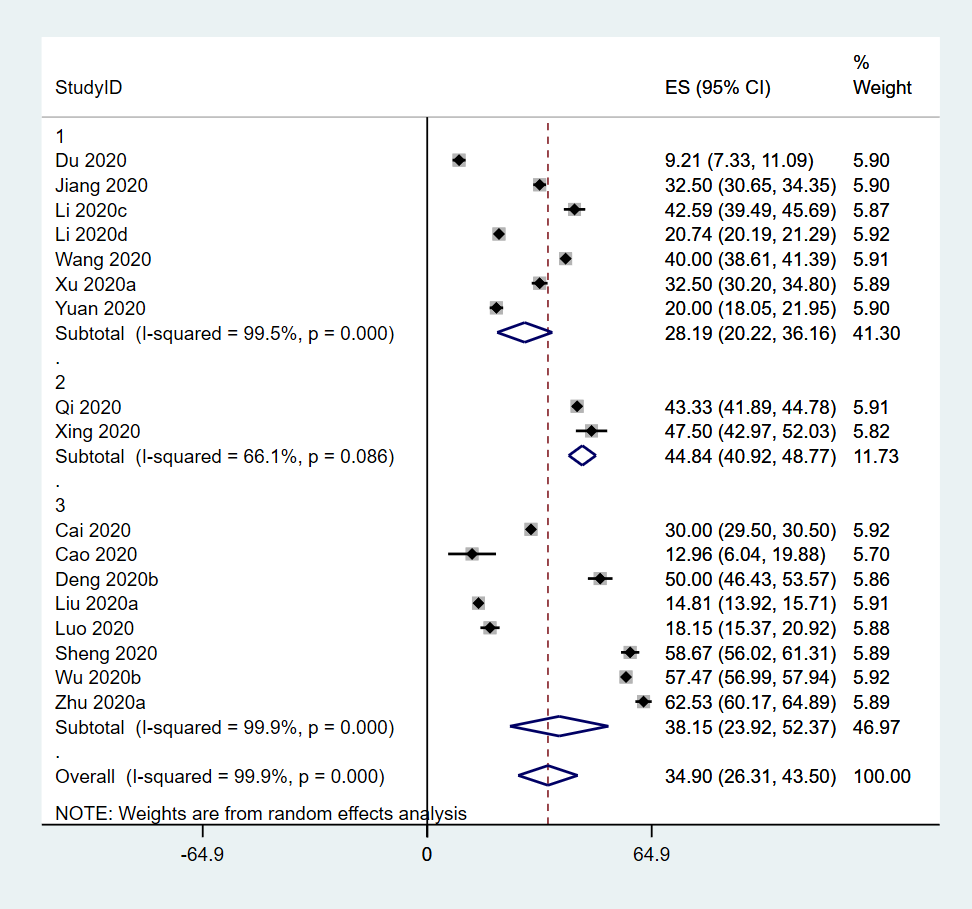


S. Fig.28 the severity of depression of HCW in Wuhan vs. Hubei province vs. other provinces in China


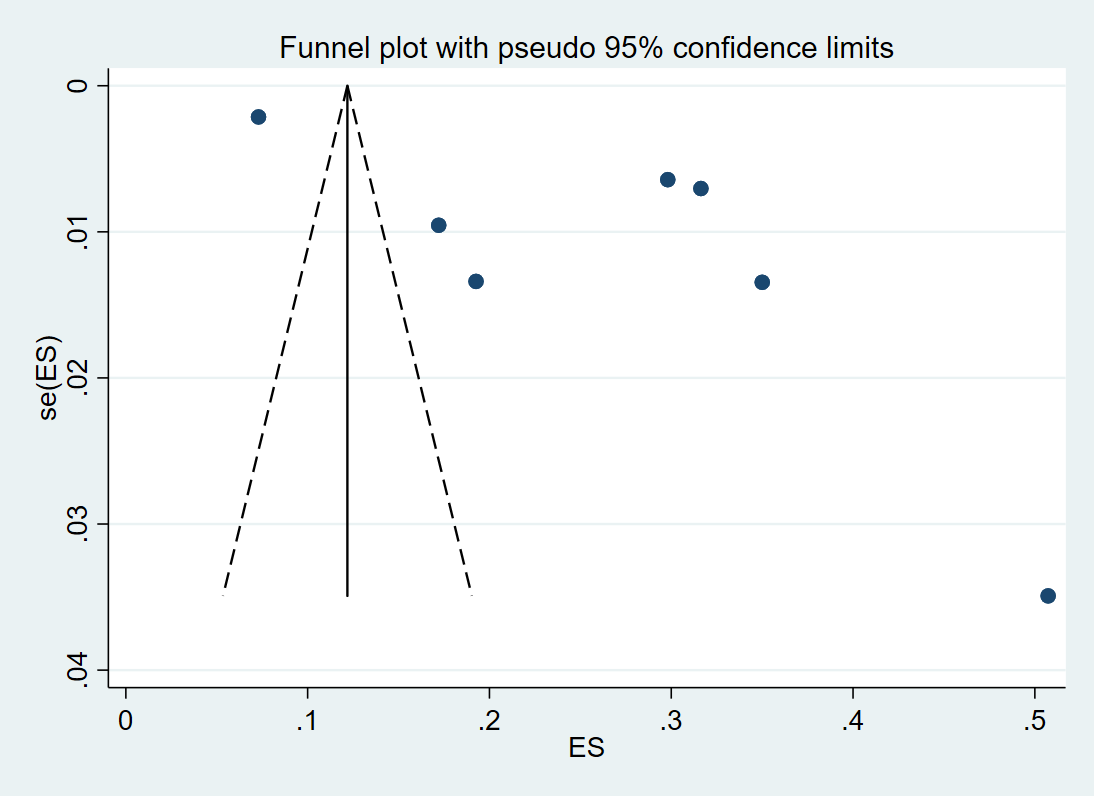


S. Fig. 29 the funnel plot of the prevalence of moderate to severe PTSS in the whole sample


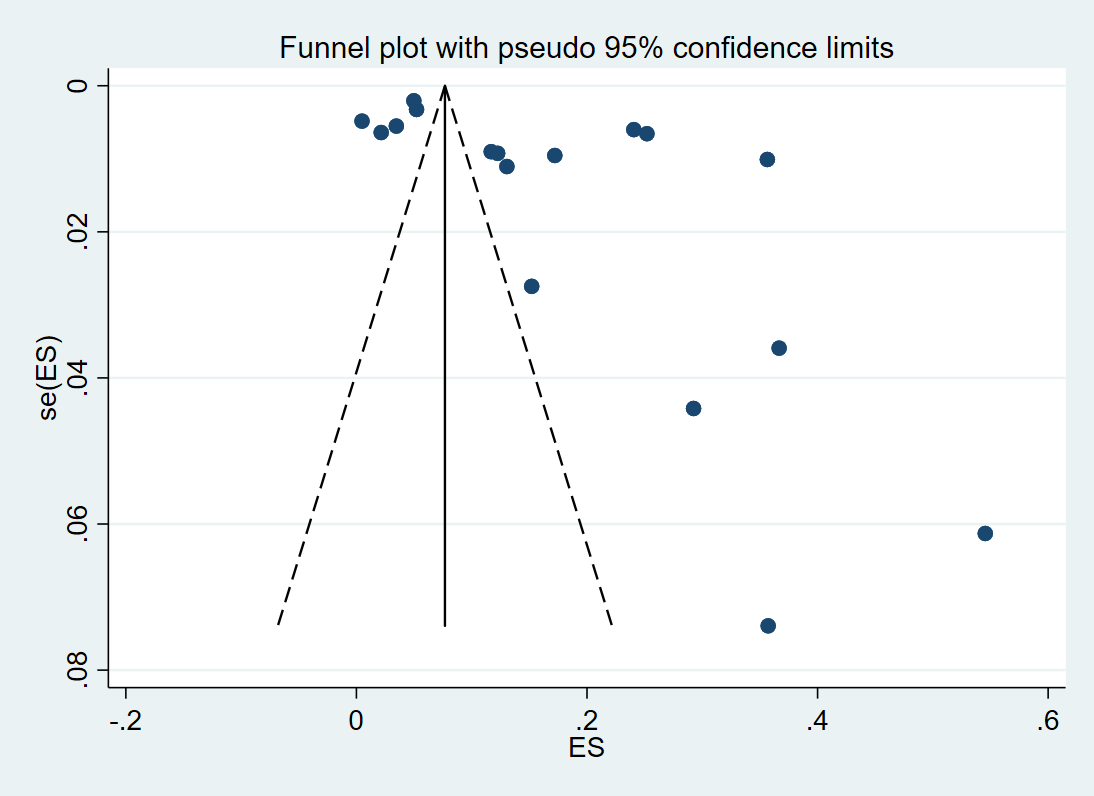


S. Fig. 30 the funnel plot of the prevalence of moderate to severe anxiety in the whole sample


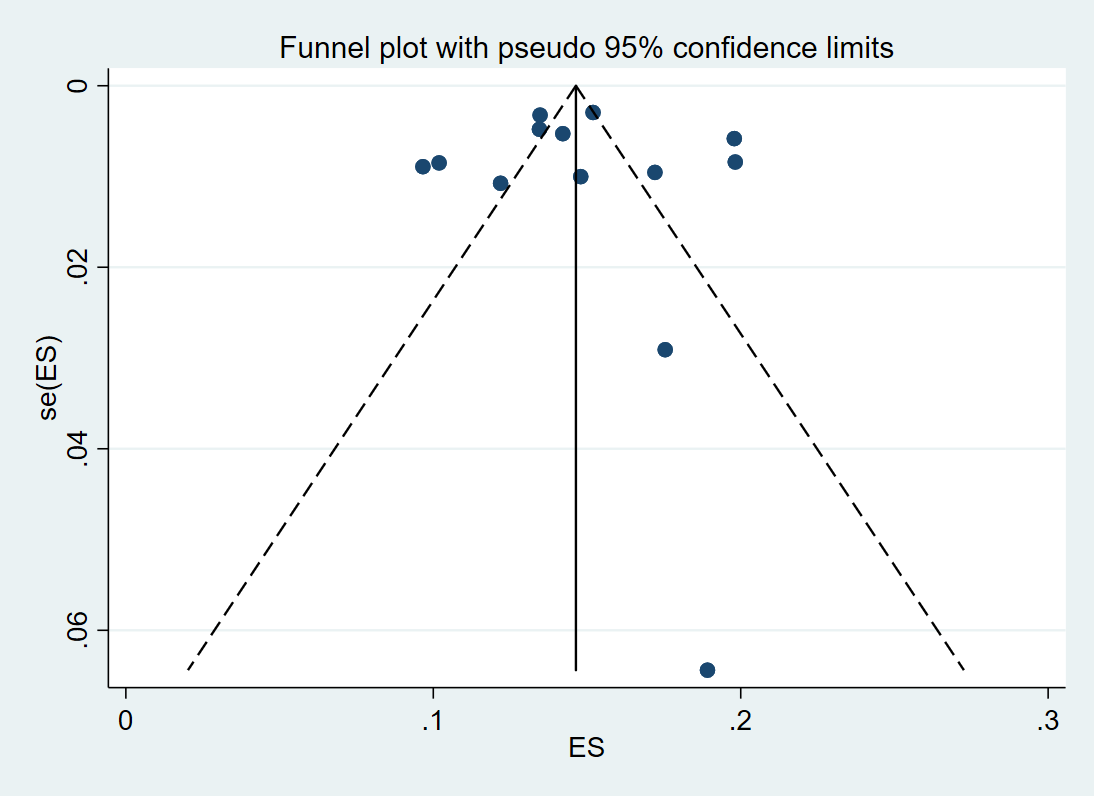


S. Fig. 31 the funnel plot of the prevalence of moderate to severe depression in the whole sample


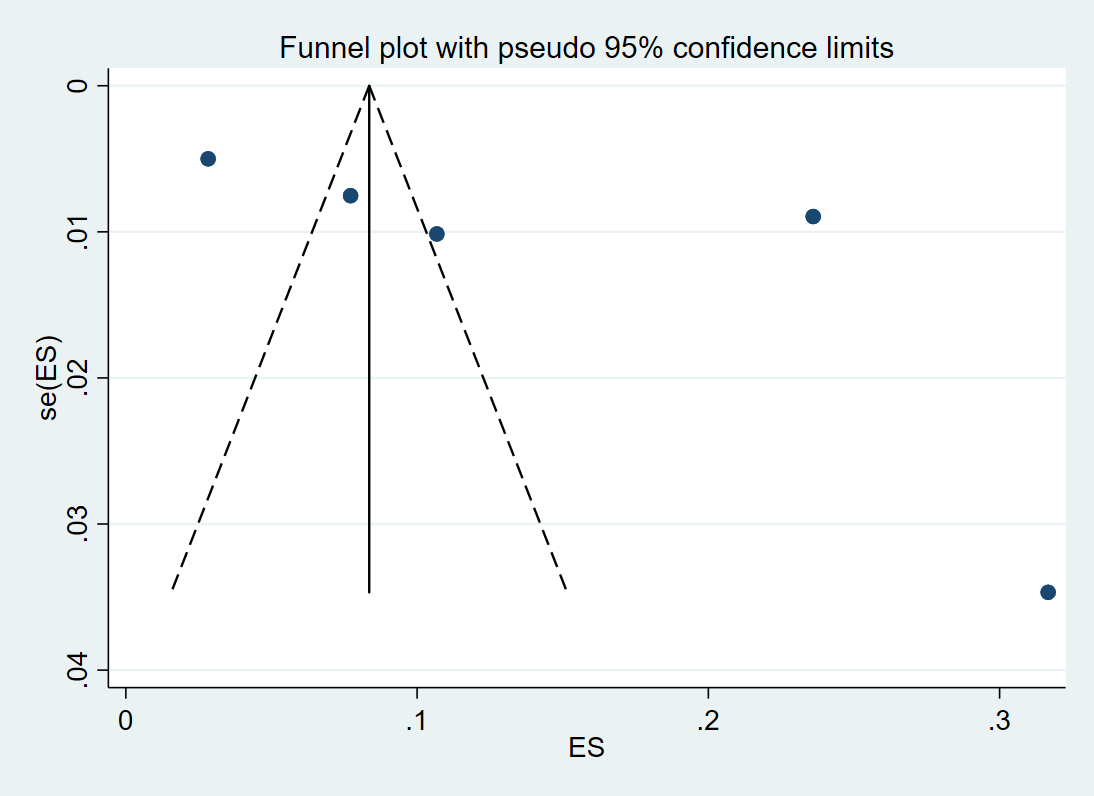


S. Fig. 32 the funnel plot of the prevalence of moderate to severe sleep disturbances in the whole sample


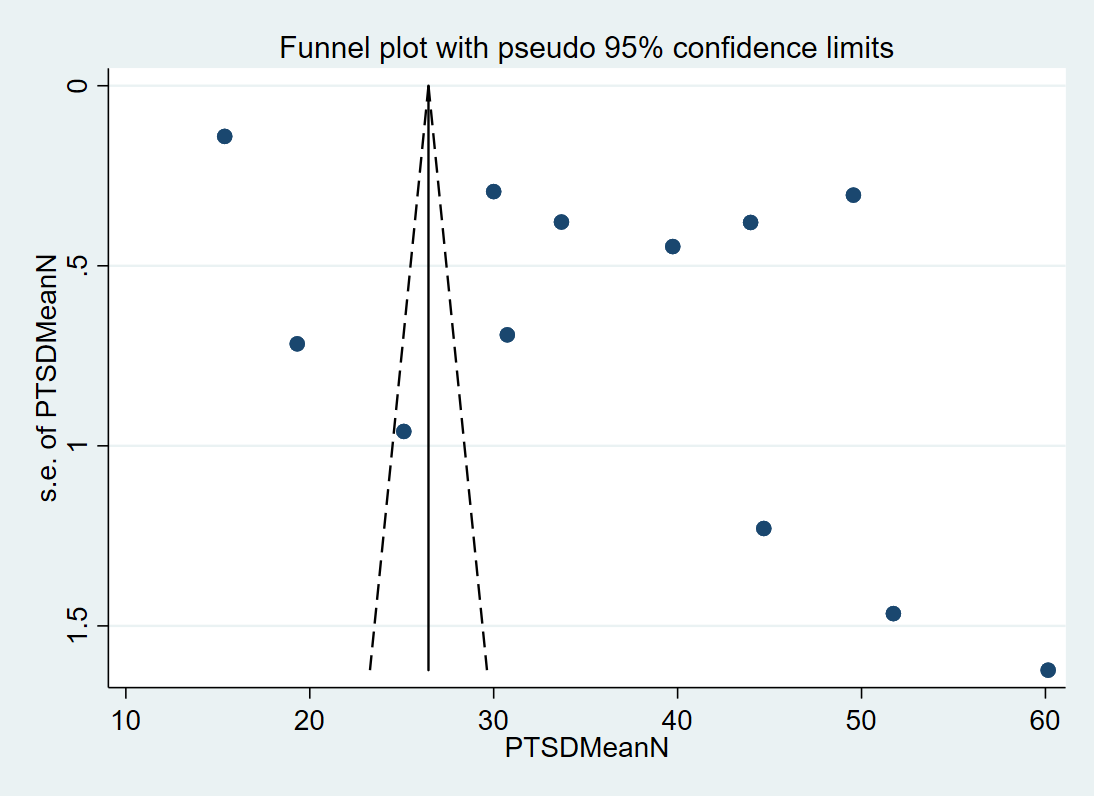


S. Fig.33 the funnel plot of the severity of PTSS in frontline and non-frontline HCW


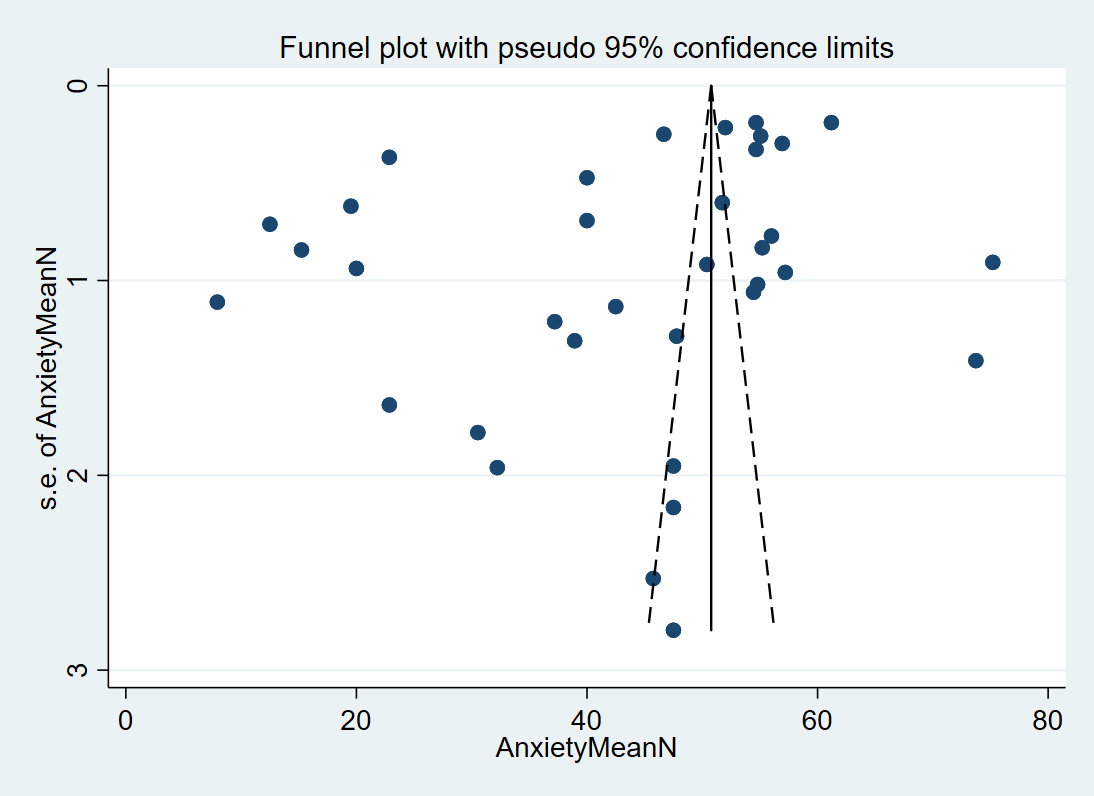


S. Fig.34 the funnel plot of the severity of anxiety in frontline and non-frontline HCW


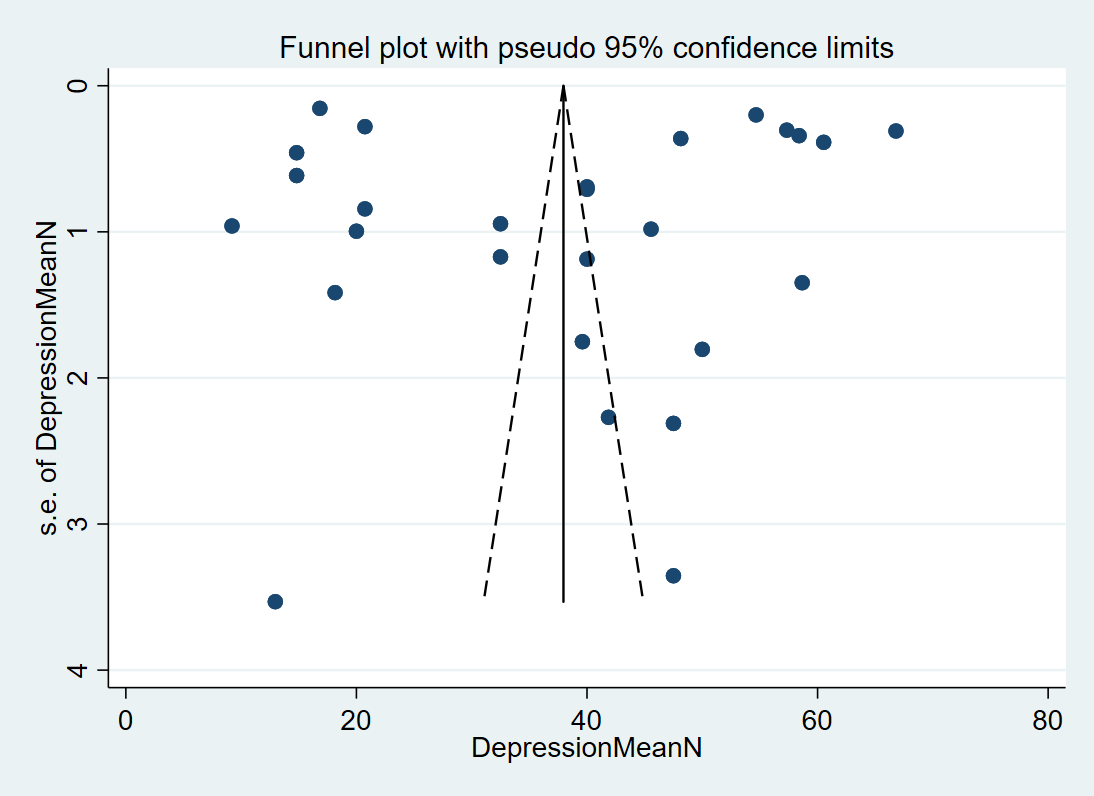


S. Fig.35 the funnel plot of the severity of depression in frontline and non-frontline HCW


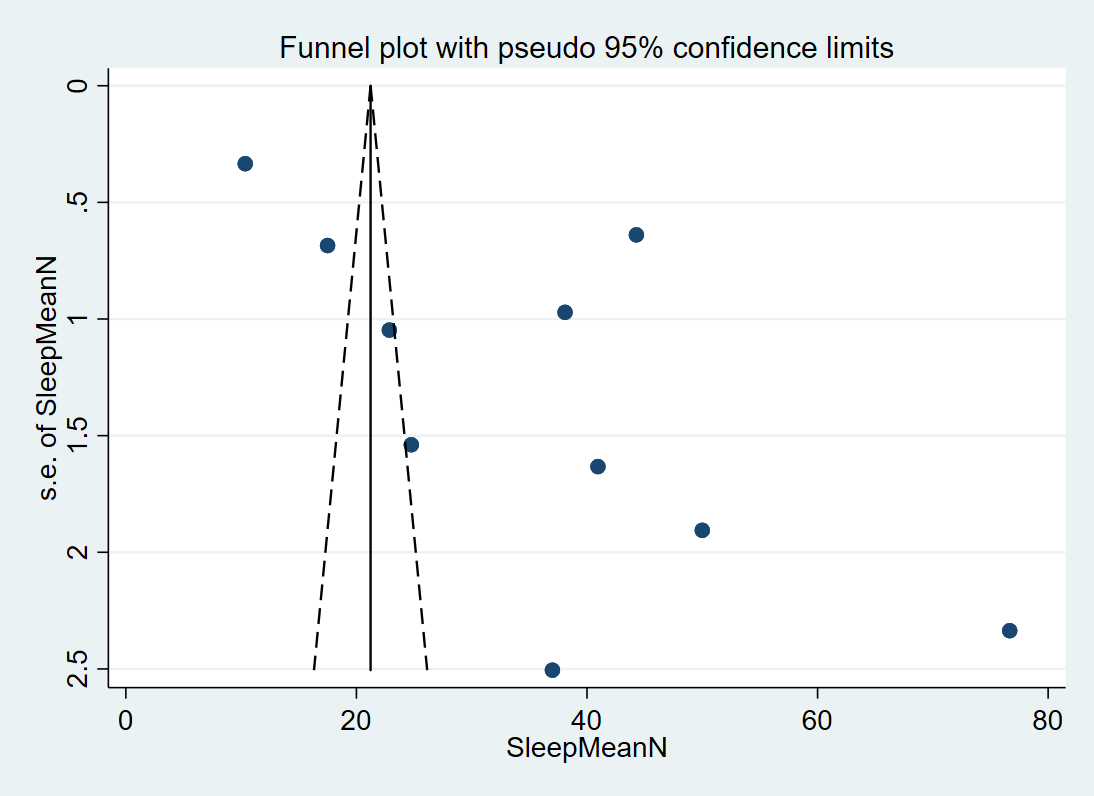


S. Fig.36 the funnel plot of the severity of sleep disturbances in frontline and non-frontline HCW
